# Evolution of Biological Hydroxyapatite Modification Strategy: Anti-Inflammation Approach Rescues the Calcium–NOD-Like Receptor–Inflammation Axis-Mediated Periodontal Redevelopment Failure

**DOI:** 10.34133/bmr.0131

**Published:** 2025-02-26

**Authors:** Xuan Zhou, Junlong Xue, Yanshu Zhang, Ruidi Xia, Zhengjie Shan, Linjun Zhang, Mixiao Gui, Guanqi Liu, Zetao Chen

**Affiliations:** ^1^ Hospital of Stomatology, Guanghua School of Stomatology, Sun Yat-sen University, and Guangdong Provincial Key Laboratory of Stomatology, Guangzhou 510055, China.; ^2^ Guangdong Research Center for Dental and Cranial Rehabilitation and Material Engineering, Guangzhou 510055, China.

## Abstract

Periodontal regenerative medicine is currently undergoing a paradigm shift from dissecting the healing process toward utilization of the developmental program. Biological hydroxyapatite (BHA), a major component of guided tissue regeneration, has long been optimized for inducing multidirectional differentiation of periodontal ligament cells (PDLCs). However, this approach runs counter to the redevelopment strategy. Thus, the conventional BHA should evolve to induce the redevelopment process of periodontal tissue. In this study, histopathological changes and immune microenvironment characteristics of the periodontal developmental process, natural healing process (Blank group), and BHA-mediated healing process (BHA group) were compared to evaluate the main manifestations of BHA-mediated periodontal “developmental engineering” outcome. Our results suggested that neither the Blank nor BHA group could recur key events in periodontal development. The implantation of BHA led to pro-inflammatory immune microenvironment and an unstable blood fibrin clot structure, which facilitated the invasion of outer gingival fibroblasts, consequently disrupting redevelopmental events. High-throughput chip technology was further used to explore the underlying mechanism of immune activation, revealing that the calcium–NOD-like receptor–inflammation axis signaling axis promoted the activation of pro-inflammatory immune response that contributed to redevelopment failure. An immunomodulatory cytokine interleukin 4 (IL4)-modified BHA was used to further validate the efficacy of developmental engineering strategy. IL4 could partially rescued the redevelopment failure through immunosuppression. This study presented an innovative strategy for the development of advanced periodontal regenerative materials and offered a potential approach for the application of development-inspired periodontal tissue engineering strategies. It represented a marked advancement in the development of regenerative medicine and propelled the clinical organ restoration forward.

## Introduction

The periodontal tissues are specialized structures that support the maintenance of teeth and withstand the forces of mastication. Achieving the ultimate goal of periodontal complex regeneration requires the regeneration of the cementum, alveolar bone, and functionally oriented periodontal ligament fibers that are properly integrated. It remains a challenge to achieve real periodontal regeneration in clinical practice, although much effort has been devoted [1]. With the evolution of regenerative medicine, innovative strategy of creating microenvironment that mimics developmental processes, which promotes cell regenerative behaviors, thereby facilitating organ regeneration, namely, “developmental engineering”, has been proposed and appreciated. This approach has shown promise in bone and tooth regeneration [2,3]. Periodontal ligament cells (PDLCs), with the capability of osteogenesis, fibrogenesis, and odontogenesis, are considered key effector cells in periodontal regeneration [4]. Our previous research has demonstrated that the redevelopmental potential of PDLCs could be partially initiated by a macrophage-mediated immune microenvironment [5,6]. These findings collectively indicated a substantial advancement in the field of periodontal regeneration research. It suggested that tissue regeneration strategies guided by the principles of periodontal development hold great potential as a viable and ultimate approach for achieving multi-tissue regeneration in the periodontium [7–9]. Consequently, a potentially effective developmental engineering approach for periodontal regeneration is urgently needed.

Bone substitutes, such as biological hydroxyapatite (BHA), are extensively utilized in clinical periodontal regeneration [10–12]. Their widespread use is primarily attributed to their capacity to promote bone tissue regeneration, which typically leads to the regeneration of bone tissue. However, the efficacy of these substitutes in regenerating the entire periodontal complex remains uncertain [13]. To enhance periodontal regeneration outcomes, there has been increasing research on modifying BHA [14–16]. Most of these studies focus on improving the regenerative properties of BHA for periodontal ligament and cementum, such as through the addition of platelet-rich fibrin [17,18] or the preparation of multilayer composite materials [19]. The underlying rationale for these modification strategies is to enhance the multidirectional differentiation capacity of effector cells. However, this approach diverges from the philosophy principles of periodontal developmental engineering. As the paradigm of periodontal regeneration has shifted toward inducing periodontal development [5], the strategy for modifying BHA should evolve to harness or recur the developmental process to facilitate periodontal regeneration. It is therefore crucial to further investigate and address questions such as whether BHA has the ability to create a developmental microenvironment and induce a redevelopmental state of cells, as well as how to modify BHA to make it suitable for developmental engineering-based periodontal restoration.

During embryonic development, the fetal immune system evades maternal immune surveillance through mechanisms such as antigenic loss, absence of classical human leukocyte antigen-I (HLA-I) molecules, and production of immunosuppressive cytokines, thereby creating an immune “buffer” zone that facilitates the development of multiple tissues [20–22]. This is a mild immune microenvironment that supports tissue development. In contrast, in adult individuals, the immune system regulates bodily functions, and the tissue repair process is under the surveillance of the immune system [23,24]. The healing process is self-limiting, involving tissue repair rather than tissue regeneration. Evidently, the functional characteristics of the immune system undergo substantial alterations during the transition from development to maturity [25]. It has been established that the tissue repair process is effectively modulated by the immune microenvironment [23,24]. However, the precise regulatory mechanisms and the underlying principles governing this regulation remain incompletely understood. This gap in knowledge presents a huge challenge for the clinical application of immune regulation in periodontal treatment. Nonetheless, from a developmental perspective, these observations appear to be both logical and substantiated. Thus, reprogramming the immune microenvironment to a developmental state may represent a crucial strategy for achieving periodontal regeneration based on developmental engineering strategies. This concept should be considered a substantial orientation for the modification of BHA.

In this study, BHA was successfully prepared (Fig. S1) and implanted into rat periodontal defect model. Then, we compared the histopathological changes and immune microenvironment characteristics of periodontal tissues during rat periodontium development, natural healing of rat periodontal defects, and the healing process following BHA implantation. We identified the key factors contributing to the failure of BHA-mediated periodontal developmental engineering. Additionally, high-throughput chip technology was performed to investigate the immune mechanisms underlying BHA’s failure to induce periodontal redevelopment. Subsequently, we modified BHA and validated its efficacy in promoting periodontal developmental engineering in rats. This study represents an important advancement in the conceptualization of BHA modification based on the development-inspired periodontal tissue engineering strategies. Our findings provide valuable insights and potential modification approaches for the broader application of developmental engineering-mediated tissue regeneration, ultimately contributing to the advancement of clinical organ restoration.

## Materials and Methods

### Preparation and characterization of BHA and IL4-BHA particles

Biological hydroxyapatite (BHA) was prepared according to our previous studies [26,27]. In brief, cancellous bone was harvested from the porcine femoral epiphysis and immersed in 30% H_2_O_2_ and anhydrous ethanol for 24 h to remove soft tissues. The bone was dissected into blocks, and the muffle furnace (SGM6812BK, Sigma Furnace Industry, China) was used to calcine at 800 °C for 2 h. Those bone blocks were grinded into particles and filtered by screen with 0.2-mm and 0.5-mm hole size to obtain 0.2- to 0.5-mm BHA particles. For interleukin 4 (IL4)–BHA, 2 mg/ml polydopamine (PDA) solution was obtained from dopamine hydrochloride (Sigma, USA) dissolved in 0.01 M Tris-base solution (Sigma, USA). Then, BHA particles were immersed in the PDA solution for 48 h and IL4 (500 ng/ml, R&D Systems, USA) solution for 24 h at 4 °C successively. The surface morphological characteristics of BHA and IL4-BHA particles were observed by modular stereo microscope (MZ10 F, Leica Microsystems, Germany) and scanning electron microscopy (SEM) (JEOL, Japan). X-ray diffraction (XRD) (Empyrean, Malvern Panalytical, Netherlands), Fourier transform infrared (FTIR) (Vertex70-Hyperion3000, Brucker, USA), x-ray photoelectron spectroscopy (XPS) (ESCALAB 250, Thermo Fisher Scientific, USA), and energy-dispersive spectroscopy (EDS) (JEOL, Japan) were used to characterize the particles. Immunofluorescence staining was used to visualize the immobilization of IL4 under a fluorescence microscope (Axio Observer Z1M, Zeiss, Germany) with eFluor 660 IL4 monoclonal antibody.

### Animal surgery and sampling

The animal surgical protocols were approved by the Institutional Animal Care and Use Committee of Sun Yat-sen University (SYSU-IACUC-2023-001018). To observe the developmental process of the periodontal complex, male Sprague–Dawley rats aged 10 d, 15 d, 25 d, and 2 months were selected and the mandibles were then collected for further analysis (*N* = 6). To observe the repair process of the periodontal complex, male Sprague–Dawley rats aged 6 weeks were selected. The periodontal defect model was prepared according to our previous studies [6]. Briefly, a mandible defect (5 × 2 × 1 mm) was prepared on the rat’s posterior tooth root area on the buccal alveolar bone (*N* = 6). The blood clot (Blank group), blood prefabricated BHA (BHA group), and blood prefabricated IL4-BHA (IL4-BHA group) were implanted in the periodontal defect area. The animals were sacrificed at 3, 7, 14, and 28 d after surgery for further analysis. At 28 d, the mandibles were scanned with a micro-computer tomography scanner (μCT50; SCANCO Medical AG, Switzerland).

### Histological analysis

The mandibles were decalcified in 10% EDTA solution for 4 weeks. For hematoxylin and eosin (H&E) staining, the nuclei were stained with Mayer’s hematoxylin (Servicebio, China), and the cytoplasm was stained with eosin (Servicebio, China). For picrosirius red staining (Servicebio, China), the slides were stained for 10 min. For immunofluorescence staining, the slides were blocked with a 5% bovine serum albumin (BSA) (Macklin, China) solution for 1 h and incubated with COL1 (1:500, Abcam, UK) and COL3 (1:100, Santa Cruz Biotechnology, USA) overnight at 4 °C. Then, the slides were incubated with goat anti-rabbit secondary antibody (1:200, Beyotime, China) and goat anti-mouse secondary antibody (1:200, Beyotime, China), while the nuclei were stained using 2-(4-amidinophenyl)-6-indolecarbamidine dihydrochloride (DAPI; Beyotime, China). For immunohistochemical (IHC) staining, the slides were blocked with a 5% BSA (Macklin, China) solution for 1 h and incubated with CD68 (1:100, Abcam, UK), mannose receptor (CD206, 1:10,000, Abcam, UK), C–C chemokine receptor type 7 (CCR7, 1:400, Abcam, UK), smooth muscle actin (SMA, 1:200, Abcam, UK), alkaline phosphatase (ALP, 1:100, Abcam, UK), and osteopontin (OPN, 1:200, Abcam, UK) after antigen retrieval. The slides were then incubated with secondary antibody (Genetech, China) for 30 min at room temperature. Images were captured using Aperio AT2 system (Leica Aperio AT2, Germany) and laser confocal microscopy (LSM980, Zeiss, Germany). The picrosirius red-stained slides were photographed using a polarized microscope (MF43, Mshot, China). Semiquantification was conducted using ImageJ software and Imagescope software.

### Characterization of blood fibrin clot

BHA was mixed with whole blood to obtain BHA fabricated blood clot. For plasma preparation, whole blood was collected in an anticoagulant tube and centrifuge for 10 min at 3,000 rpm. The BHA was mixed with obtained plasma and thrombin reagent (Haifei, China) for 10 min to obtain BHA fabricated fibrin clot. The clots were soaked in a 2.5% glutaraldehyde solution, followed by graded ethanol, solvent replacement, and lysophilization, and then characterized by SEM (JEOL, Japan). The porosity of fibrin networks was measured using ImageJ software. Protein concentration in normal plasma and BHA-coworked plasma was detected by bicinchoninic acid assay (BCA protein assay, Cwbiotech, China). Activated partial thromboplastin time (APTT), prothrombin time (PT), thrombin time (TT), fibrinogen (FIB), and the concentration of clotting factors were measured (KingMed Diagnostics, China). Calcium ion concentration was also detected (Agilent 720ES, USA).

### Fibrin clot preparation and macrophage culture

Normal blood and BHA-coworked blood collected from male Sprague–Dawley rats was placed in a 6-well cell culture plate and soaked in red blood cell lysis buffer to obtain fibrin clots. Dulbecco’s modified Eagle’s medium (DMEM; Gibco, USA) with 10% fetal bovine serum (Gibco) and 1% penicillin/streptomycin (Gibco) was used as the medium to culture RAW 264.7 cell line. Macrophages were seeded on the prepared fibrin clots and cultured for 48 h. The cultured macrophages were used for RNA sequencing (RNA-seq), real-time quantitative polymerase chain reaction (RT-qPCR), and immunofluorescence staining.

### Transcriptome sequencing of macrophages and single-cell transcriptome sequencing of tissue in rat periodontal defects

RNA-seq and single-cell RNA-seq (scRNA-seq) were performed using separately the BGISEQ-500 platform at BGI (Wuhan, China) and Illumina NovaSeq 6000 platform at NovelBio (Shanghai, China). Correlation analysis, principal components analysis (PCA) , Gene Set Enrichment Analysis (GSEA) analysis, Gene Ontology (GO) and Kyoto Encyclopedia of Genes and Genomes (KEGG) analyses, and Uniform Manifold Approximation and Projection (UMAP) were performed on Dr. Tom (https://biosys.bgi.com) and CytoNavigator (https://sc.novelbrain.com). Interaction analysis and visualization were conducted by string database, Gephi (v0.9.2), and Cytoscape (v3.10.1). All data visualization was performed by GraphPad Prism 9, Sangerbox (https://vip.sangerbox.com), OmicStudio tools (https://www.omicstudio.cn/tool), ChiPlot (https://www.chiplot.online), and Gephi (v0.9.2).

### RT-qPCR and immunofluorescence staining

The extracted total RNA was detected by Nanodrop (Thermo Fisher, USA) and then converted to complementary DNA by PrimeScript RT Master Mix (Takara, Japan). Equivalent cDNA samples were analyzed by RT-qPCR using Hieff qPCR SYBR Green Master Mix (Yeasen, China) on an ABI 2-step system (Applied Biosystems, USA). Primer sequences were presented in the Supplementary Materials (Table S1). The obtained samples were calculated based on the 2^−∆∆Ct^ method referring to glyceraldehyde-3-phosphate dehydrogenase (GAPDH). For calcium ion imaging, macrophages were labeled with Fluo-4 AM (Beyotime, China). For immunofluorescence staining, macrophages were stained with CD206 (1:10,000, Abcam, UK) and inducible nitric oxide synthase (iNOS) (1:100, Santa Cruz Biotechnology, USA). Goat anti-rabbit secondary antibody (1:200, Beyotime, China), goat anti-mouse secondary antibody (1:200, Beyotime, China), and DAPI (Beyotime, China) were used. Images were captured using a fluorescence microscope (Axio Observer Z1 M, Zeiss, Germany).

### Statistical analysis

The experimental results are presented as the mean ± SD. Statistical analyses were performed using GraphPad Prism software. Student’s *t* test or one-way analysis of variance (ANOVA) analysis was applied to compare the differences between groups. The significance level (α) was set at 0.05, and statistical significance was considered for *P* values < 0.05. *P < 0.05; **P < 0.01; ***P < 0.001; ****P < 0.0001.

## Results

### Failure of BHA in inducing periodontal “developmental engineering”

**Fig. 1.**
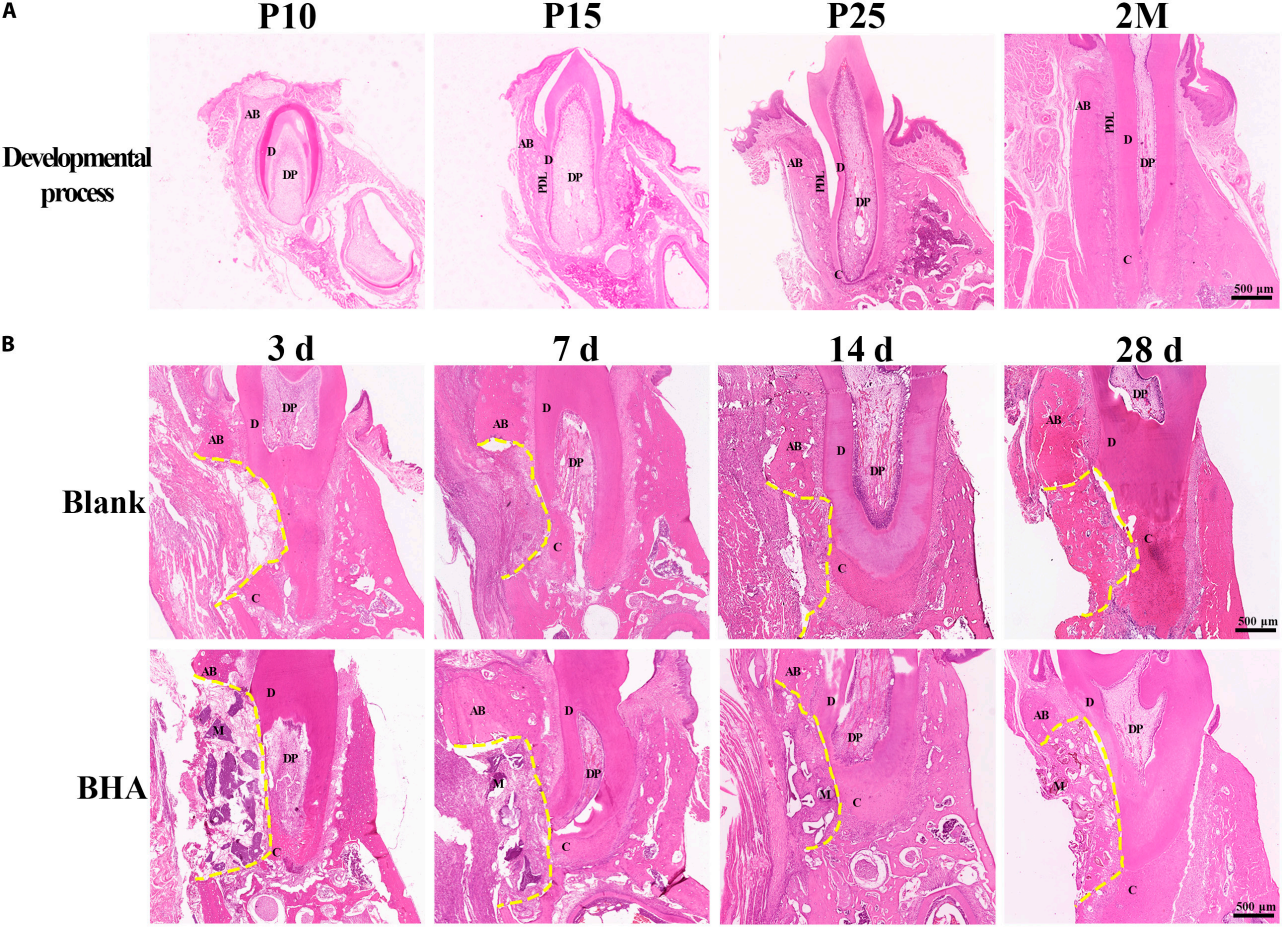
Failure of BHA in inducing periodontal “developmental engineering”. (A) Developmental stages of the rat mandible molar at postnatal days 10, 15, and 25 and 2 months. (B) Comparison of natural (Blank group) and BHA-mediated (BHA group) periodontal multi-tissue healing process at 3, 7, and 14 d and 1 month after surgery. AB, alveolar bone; D, dentin; PDL, periodontal ligament; C, cementum; DP, dental pulp; M, material; yellow line, defect area edge.

An overview of the periodontal developmental and repair process is shown in Fig. 1A. Periodontal tissue development typically originates from the dental follicle and involves key developmental signals, including epithelial induction, PDL fiber anchorage, growth, and mineralization. These processes ultimately coordinate the formation of cementum, periodontal ligament, alveolar bone, and outer gingival fibers. In both the Blank and BHA groups, the periodontal tissue failed to return to its natural structure after surgery. Fibers within the periodontal ligament area remained disarranged and detached from the dentin (Fig. 1B). The alveolar bone was not fully restored, as bone depression could be observed in the Blank group. While bone width was preserved in the BHA group, bone mineralization in the defect area remained incomplete, and the periosteal structure was still unclear. Additionally, the ingrowth of fibers from the gingival connective tissue could also be observed, potentially interfering with the bone repair process. Furthermore, no cementum regeneration could be observed and the newly formed periodontal ligament exhibited weak attachment to the root, rendering it incapable of withstanding occlusal forces.

**Fig. 2.**
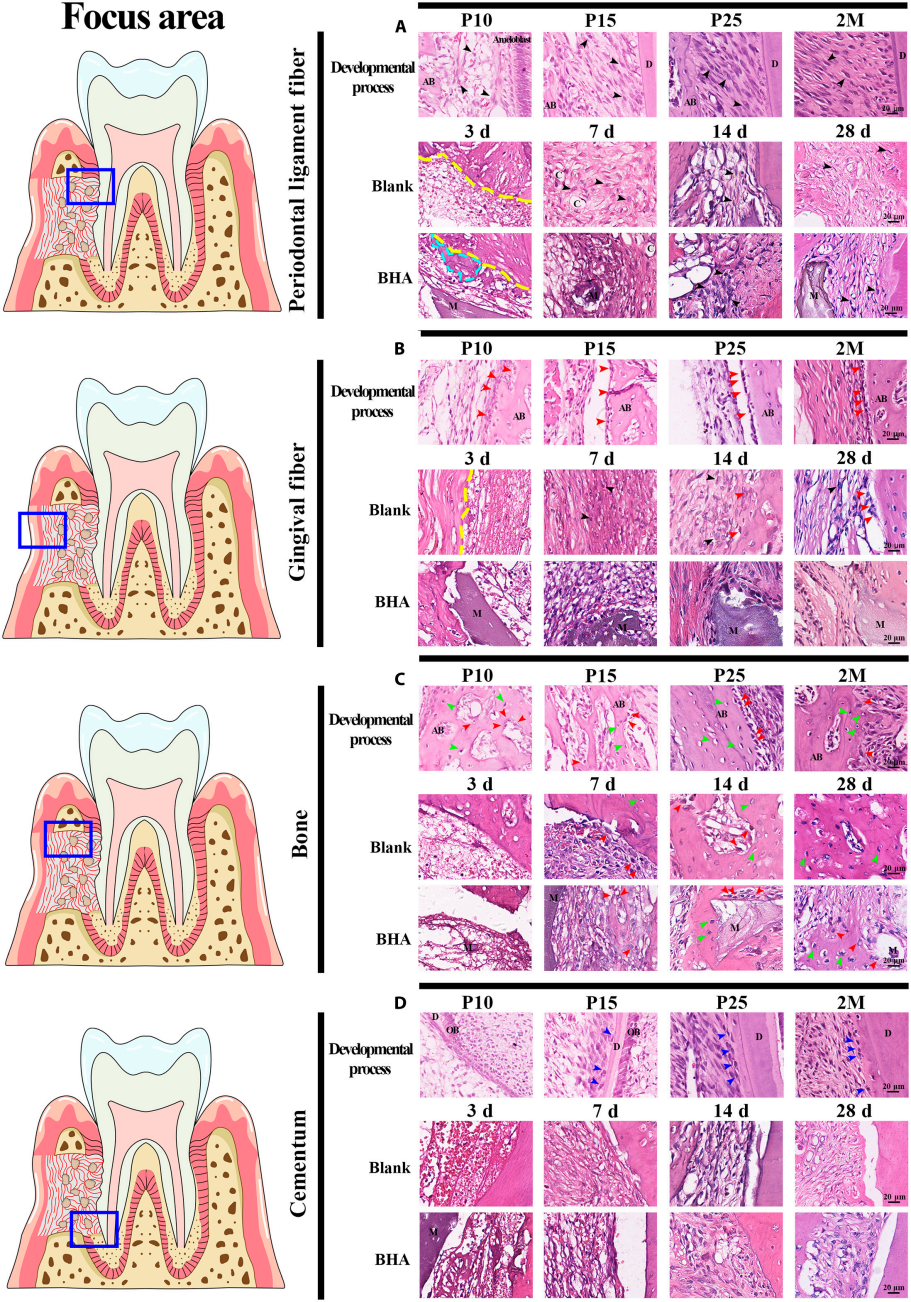
Organized periodontal tissues in the developmental process and disorganized newly formed tissues in the healing process. (A) Periodontal ligament fiber. (B) Gingival fiber. (C) Alveolar bone. (D) Cementum. Black arrowhead, fibroblast; red arrowhead, osteoblast; green arrowhead, osteocyte; blue arrowhead, cementoblast; AB, alveolar bone; C, capillary; D, dentin; M, material; OB, odontoblast; yellow line, defect area edge; light blue dotted area: fibrinoid necrosis area; blue rectangles: focus area in Blank and BHA groups.

**Fig. 3.**
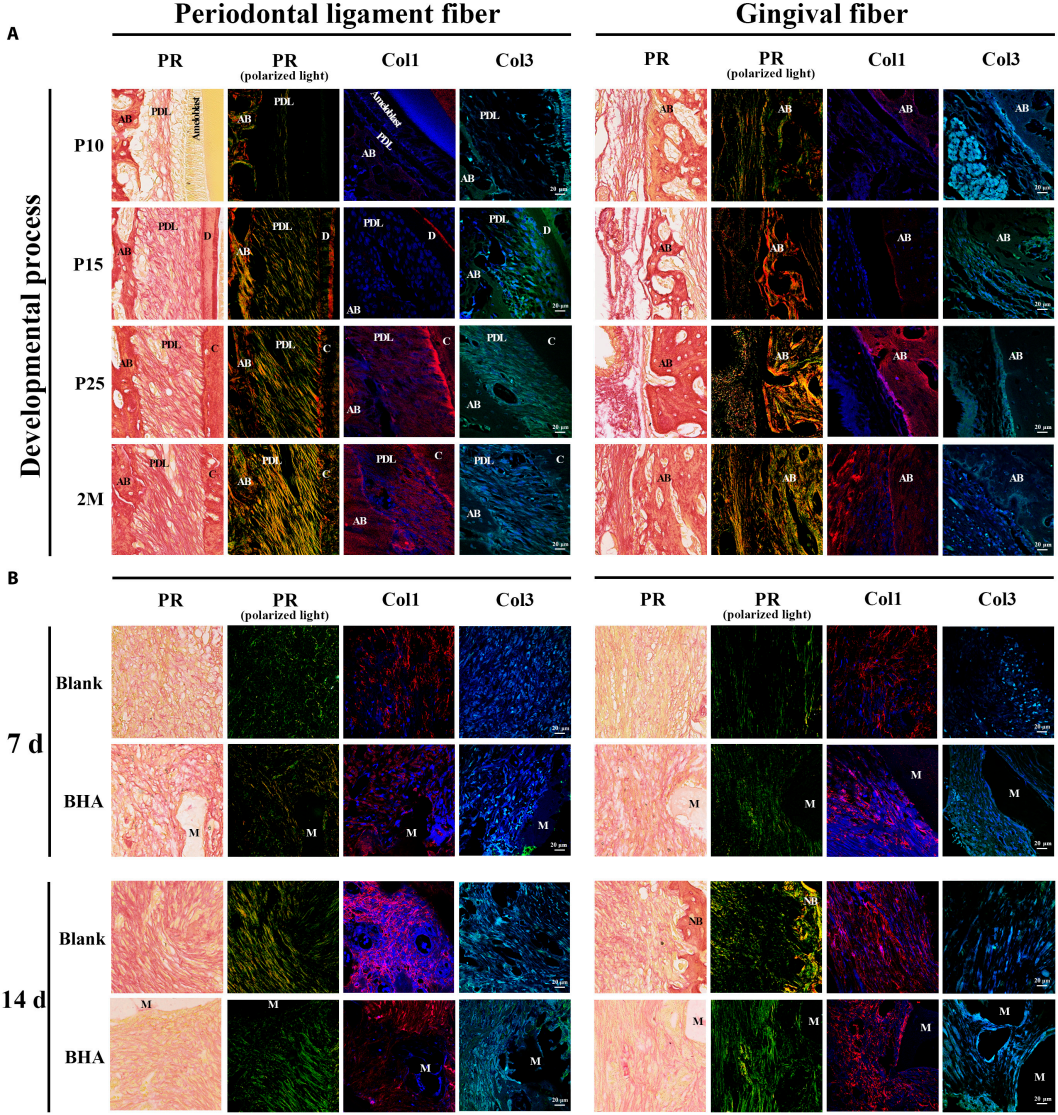
Organized ECM in the developmental process and remodeling failure of the native structure due to the interference of outer gingival fibers in the healing process. PR staining under nonpolarized/polarized light microscopy and immunofluorescent staining of Col1 and Col3 (A) in the developmental process and (B) at 7 and 14 d after surgery. Under polarized microscope, Col1 appeared as dense thick fibers with bright yellow or red color, while Col3 appeared as loose fine fibers with green color. AB, alveolar bone; D, dentin; PDL, periodontal ligament; C, cementum; M, material.

In the periodontal ligament area, the periodontal space initially developed from a loosely arranged collagenous mass containing periodontal fibroblasts (Fig. 2A). The number of fibroblasts and collagen bundles increased, forming an interconnected network. These fiber bundles, primarily composed of COL1 and COL3, gradually aligned to accommodate occlusal forces (Fig. 3 and Fig. S2). In the Blank group, the blood fibrin clot at 3 d after surgery was dense and had a thin diameter, which was subsequently replaced by granulation tissue by day 7. Fibroblasts from the periodontal ligament migrated into the defect area, proliferating actively. The enlarged nuclei of these proliferating fibroblasts indicated high functional activity, such as synthesis, migration, and division, suggesting a strong reparative potential. By day 14 after surgery, the extracellular matrix (ECM) was increasingly enriched, with elevated levels of COL1 and COL3, although it had not yet fully returned to its natural structure (Fig. 3B and Fig. S3). At 28 d after surgery, the newly formed periodontal ligament connected with the preexisting tissue, but the fiber orientation remained disorganized (Fig. 2A). In the BHA group, the migration of PDLCs appeared to be impeded by fibrinoid necrosis at the defect margin, potentially leading to a delayed periodontal tissue repair process. This delay was further reflected in the synthesis of ECM, which was reduced at 7 and 14 d after surgery, as evidenced by the lower levels of collagen production (Fig. 3B and Fig. S3). The ECM remained loosely organized even at 28 d after surgery (Fig. 2A). In summary, the periodontal ligament failed to restore its natural structure after surgery, characterized by disorganized fibers and reduced collagen content. The delayed migration of PDLCs may have also diminished the potential for developmental engineering, as PDLCs, derived from dental follicle cells, retain the capacity to replicate developmental events.

The interface between the alveolar bone and outer gingival connective tissue was maintained by the periosteal structure from the early developmental stage (Fig. 2B and C). On the periodontal ligament side, PDLCs generated new fibers that adhered to the nonmineralized matrix deposited by osteoblasts. These fibers became entrapped as the bone matrix further calcified. In the Blank group, the blood fibrin clot exhibited a surface fibrin film at the interface between the defect area and outer fibers at 3 d after surgery, which inhibited the early ingrowth of outer gingival fibers (Fig. 2B). This interface remained distinct at 7 d after surgery, hindering the migration of outer gingival fibroblasts. Cuboidal osteoblasts were observed at the defect edge during this period (Fig. 2C), indicating active bone regeneration potential. By 14 d after surgery, the boundary between bone and outer gingival fibers began to establish from the mineralized bone edge, accompanied by a reduction in bone width. In the BHA group, the surface film structure of the fibrin clot was disorganized and poorly defined at 3 d after surgery (Fig. 2C). There was no distinct demarcation between the blood clot and the outer gingival fibers, which facilitated the migration of fibroblasts from the outer gingival connective tissue into the defect area. By 7 d after surgery, the defect area was populated with osteoid tissue consisting of an organic matrix and osteoblasts. However, the area of calcification did not exhibit a significant increase at 14 and 28 d after surgery, potentially due to the encroachment of fibroblasts from the outer gingival connective tissues (Fig. 2B). The synthesis and deposition of COL1 and COL3 were elevated at both 7 and 14 d after surgery (Fig. 3), indicative of enhanced fibrotic activity. This increase in tissue fibrosis was associated with inhibited mineralization of the alveolar bone. In conclusion, the unstable structure of the fibrin clot in the BHA group facilitated the invasion of gingival connective tissue fibroblasts, disrupting natural bone tissue repair and periosteum regeneration. While BHA demonstrated an ability to promote early-stage bone regeneration, this effect was attenuated by the interference of outer gingival fibers.

Regarding cementum development, mesenchymal stem cells within the dental follicle differentiated into cementoblasts on the dentin side and subsequently synthesized a collagenous cementum matrix following the disruption of Hertwig’s epithelial root sheath (Fig. 2D). PDLCs extended numerous cytoplasmic processes that interfaced with the nonmineralized cementum, thereby establishing a connection between the root and PDL. Throughout the healing process, fibroblasts adhered to the dentin and continuously deposited collagen; however, differentiation into cementoblasts was not observed. Consequently, a successful integration of the periodontal ligament with the cementum was not achieved.

In conclusion, the blood fibrin clot structure formed during the early stages of the healing process in the BHA group was unstable, facilitating the invasion of outer gingival fibroblasts. Although BHA implantation preserved osteogenic capacity, bone mineralization and periosteal reconstruction in the defect area were disrupted. Moreover, the migration of PDLCs was hindered by fibrinoid necrosis at the defect margin, which led to delayed ECM synthesis, disorganized periodontal fiber arrangement, and impaired differentiation into cementoblasts, ultimately resulting in weak attachment to the tooth root. Consequently, the BHA group failed to recur critical events in periodontal development, such as PDL fiber anchorage and growth, normal alveolar bone, and dental bone mineralization. As a result, it could not achieve true multi-tissue regeneration of the periodontium based on development-inspired engineering strategies.

### Failure of BHA to induce “developmental engineering” results from a macrophage-mediated pro-inflammatory microenvironment

As mentioned above, the immune microenvironment may be a critical factor in developmental engineering. Recent studies have highlighted the important role of macrophages in tissue development, maturation, and the healing process during both developmental and adult stages [22]. Our previous research has also demonstrated the “2-sided coin” effect of the macrophage-mediated immune microenvironment [5,6,28]. Therefore, the role of macrophage-mediated immune microenvironments during both development and healing was further analyzed.

**Fig. 4.**
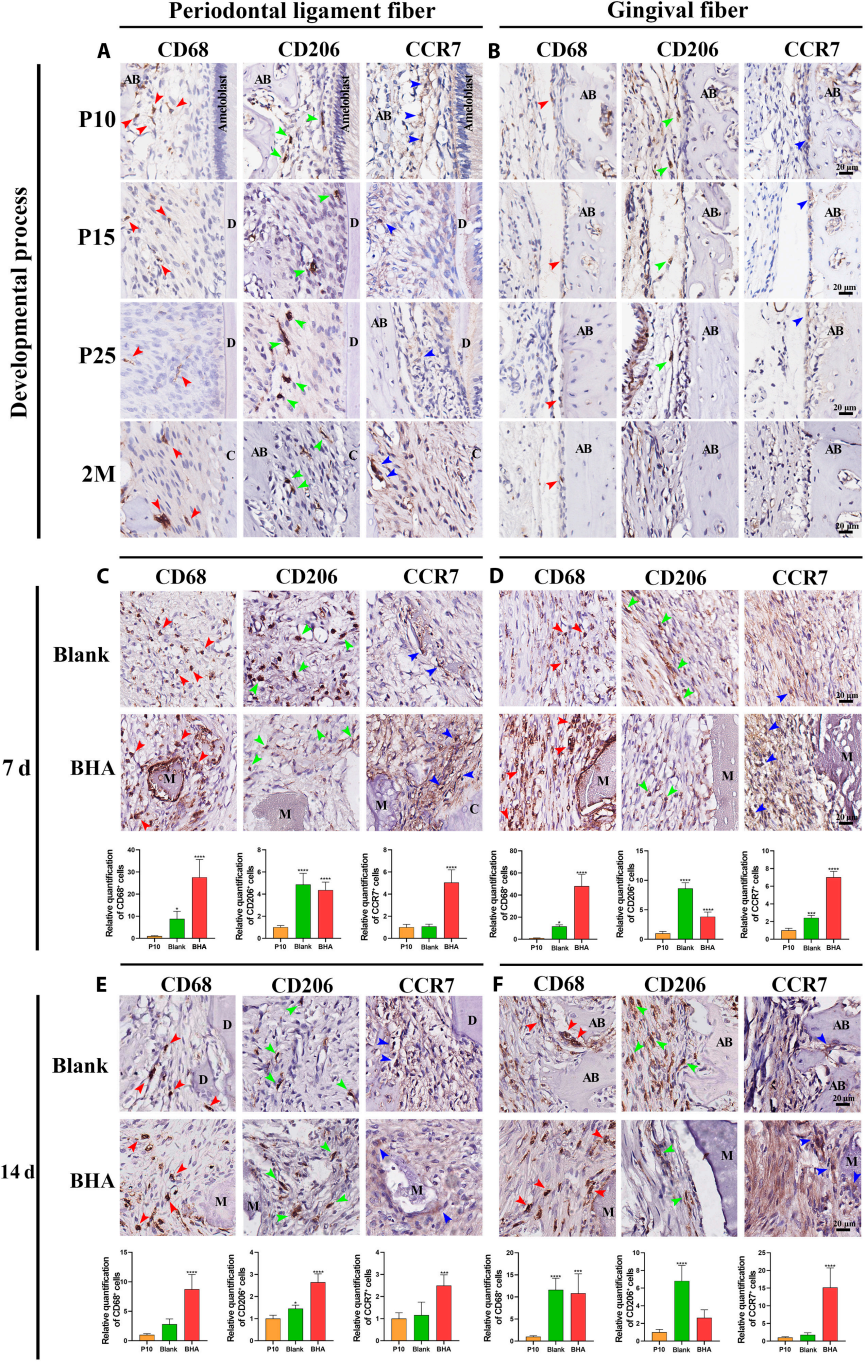
Involvement of immune cells in the developmental process and the overactivated pro-inflammatory immune microenvironment during the BHA-mediated healing process. (A and B) IHC staining image of macrophage markers (CD68, CD206, and CCR7) during the developmental process. (C to F) IHC staining image and quantitative analysis of the CD68^+^, CD206^+^, and CCR7^+^ cells at 7 and 14 d after surgery. AB, alveolar bone; D, dentin; PDL, periodontal ligament; C, cementum; M, material; red arrowhead, CD68^+^ cells; green arrowhead, CD206^+^ cells; blue arrowhead, CCR7^+^ cells. *P < 0.05; **P < 0.01; ***P < 0.001, ****P < 0.0001.

During the developmental process, CD68-positive (a pan-macrophage marker), CCR7-positive (an M1 macrophage marker), and CD206-positive (an M2 macrophage marker) cells were observed in the periodontal ligament and outer gingival fiber areas, albeit in small numbers (Fig. 4A and B). These results confirmed the involvement of immune cells in the developmental process, where they play a crucial role in maintaining tissue homeostasis [29]. During the healing process, the immune response was activated as early as the hemostasis stage, markedly regulating subsequent repair events. The number of CD68-positive cells increased significantly in both the Blank and BHA groups at 7 and 14 d after surgery, indicating a strong activation of the immune response. This suggested that the immune microenvironment during healing is distinctly different from that of the developmental stage. In the periodontal ligament area, the BHA group also exhibited a pro-inflammatory immune microenvironment at 7 d after surgery, characterized by a decreased number of CD206-positive cells and increased number of CCR7-positive cells (Fig. 4C). By 14 d after surgery, the number of CD206-positive cells in the BHA group had increased, suggesting a gradual attenuation of the immune response and a shift toward an M2-like microenvironment. This M2-like microenvironment could stimulate fibroblast ingrowth, proliferation, ECM synthesis, and tissue remolding [30], thus avoiding excessive fiber deposition and fibrosis activity. The rapid transition of the immune microenvironment in the periodontal ligament area may be attributed to the involvement of PDLCs, which possess immunoregulatory functions and can modulate the inflammatory response. Conversely, in the periosteum and outer gingival fiber areas, the number of CD206-positive cells decreased, while the number of CCR7-positive cells increased in the BHA group at 7 d after surgery, indicating an enhanced pro-inflammatory response. This pro-inflammatory immune microenvironment persisted through 14 d after surgery (Fig. 4E and F). Furthermore, the pro-inflammatory response was more pronounced in these areas compared to the periodontal ligament area. The sustained pro-inflammatory microenvironment could lead to a fibrotic response in fibroblasts, promoting the excessive production of ECM components, including COL1 and COL3 [31,32].

In summary, the macrophage-mediated immune microenvironment exhibited significant differences between the developmental and healing processes. During the BHA-mediated healing process, an excessively activated pro-inflammatory immune microenvironment driven by macrophages was observed, which inhibited the proliferation and differentiation of PDLCs, thereby limiting their potential to recur the periodontal developmental state. Additionally, this pro-inflammatory environment promoted the proliferation of outer gingival fibroblasts and the production of ECM, leading to a deleterious fibrotic response and subsequent failure of developmental engineering.

### The pro-inflammatory microenvironment generated during BHA-mediated processes inhibits periodontal developmental engineering by activating the calcium–NOD-like receptor–inflammation axis

Since the fibrin clot was considered as the initiation of the immune microenvironment [6], the impact of BHA on fibrin clot formation and macrophages seeded on the fibrin clot was analyzed via RNA sequencing (RNA-seq) to investigate the mechanism of macrophage activation in the BHA group.

**Fig. 5.**
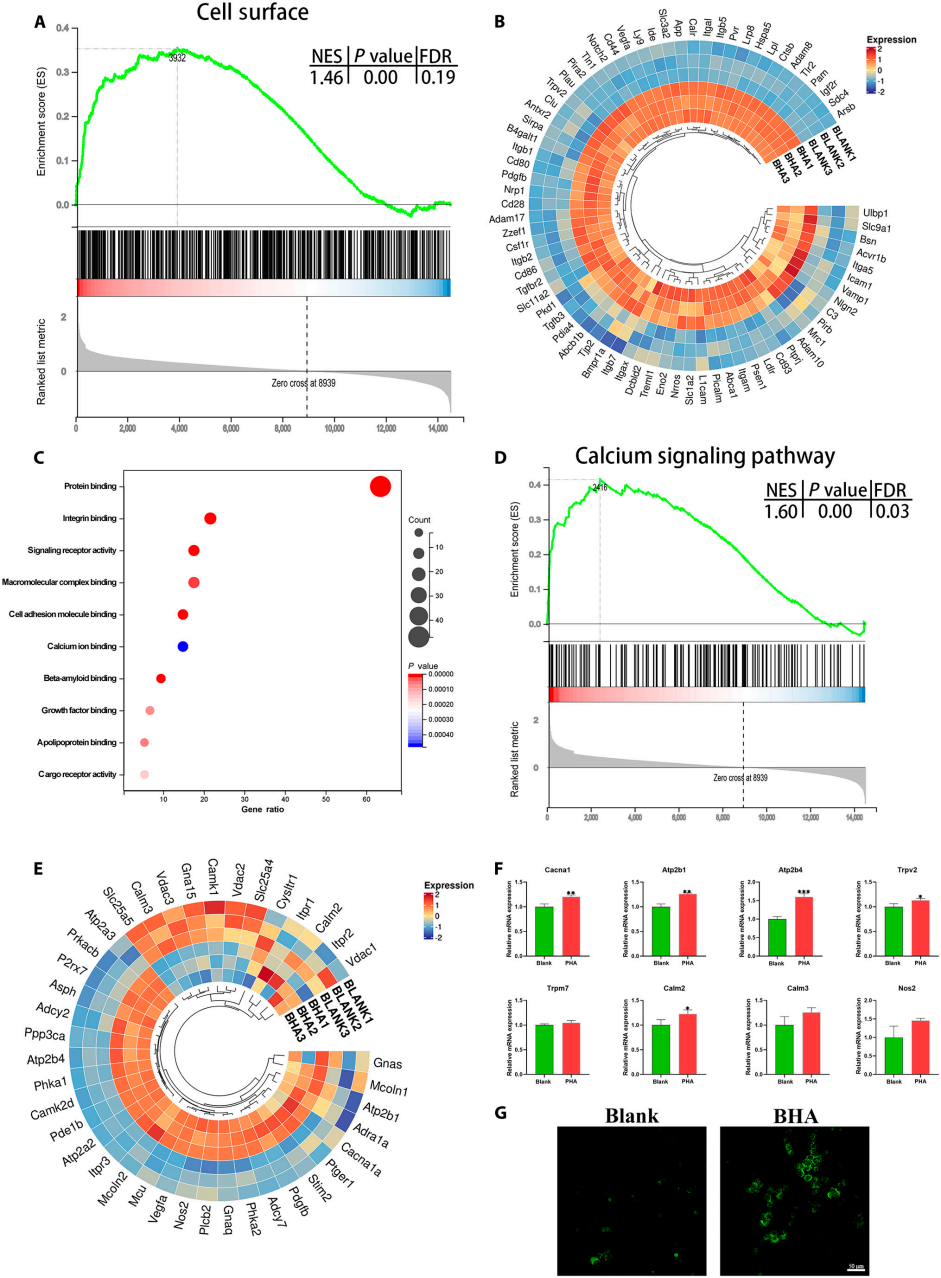
The calcium signaling pathway of macrophage was activated in the BHA group. (A) GSEA result of cell surface term enriched. |Normalized enrichment score (NES)| > 1, *P* < 0.05, and false discovery rate (FDR) *q* < 0.25 are considered significant. (B) Differentially expressed genes in cell surface term. *P* values of <0.05 are considered significant. (C) GO Molecular Function analysis of differentially expressed genes in cell surface term. (D) GSEA result of KEGG analysis of the calcium signaling pathway. (E) Heatmap of differentially expressed genes in the calcium signaling pathway. (F) RT-qPCR result of calcium signaling pathway-related genes. (G) Detection of Ca^2+^ by fluorescence microscopy. *P < 0.05; **P < 0.01; ***P < 0.001.

As shown in Fig. S4A, the fibrin clot structure in the BHA group was looser and exhibited greater porosity compared to the normal blood clot structure. This result was consistent with in vivo findings. BHA could regulate fibrin clot formation by adsorbing blood proteins involved in the intrinsic coagulation pathway (factors XII, XI, IX, and VII), the extrinsic coagulation pathway (factor VII), and the common coagulation pathway (factors X and II), as well as by releasing Ca^2+^ (Fig. S4B to E). The fibrin clot has multiple Ca^2+^ binding sites, suggesting an increased amount of Ca^2+^ bound in the BHA group fibrin clot [33]. Correlation analysis and PCA suggested high intra-group reproducibility and significant differences between the Blank and BHA groups (Fig. S5A and B). Differentially expressed genes were shown in Fig. S5C. The top 10 enriched up-regulated GO terms including Cellular Component, Biological Process, and Molecular Function were shown in Fig. S5D. Among all the up-regulated GO terms, the “cell surface” term involved the highest number of genes (Fig. 5A and B). The altered fibrin clot structure may initially regulate macrophages via cell surface receptors. Consequently, differentially expressed genes related to cell surface were subjected to GO Molecular Function analysis (Fig. 5B), revealing an enrichment of the “Calcium ion binding” term (Fig. 5C). The GSEA result from the KEGG database further confirmed this finding (Fig. 5D). Moreover, most of the genes involved in the calcium signaling pathway were up-regulated in the BHA group (Fig. 5E), which was further validated by RT-qPCR results and fluorescence staining of Ca^2+^ (Fig. 5F and G). Collectively, these results suggested that the calcium signaling pathway was a major activated pathway when macrophages interact with the fibrin clot. As mentioned above, the increased Ca^2+^ binding in the BHA group fibrin clot likely activated the calcium signaling pathway. Macrophages could recognize the N terminus of the fibrin α-chain and rapidly internalize fibrin monomers via lysosomes [34,35]. With the increased Ca^2+^ bound to the fibrin clot, more Ca^2+^ is likely released. Additionally, the instability of the fibrin clot structure may also result in its rapid collapse, further promoting Ca^2+^ release and subsequently activating the calcium signaling pathway.

Endocytosed Ca^2+^ can activate the NOD-like receptor (NLR) signaling pathway, which has been widely reported [36–39]. Ca^2+^ can be derived not only from the extracellular environment but also from the endoplasmic reticulum (ER) and lysosomal compartments. Inhibition of Ca^2+^ channels can substantially hinder the activation of inflammatory factors such as caspase-1 and IL1β [40]. Additionally, increased extracellular Ca^2+^ may interact with the serum protein fetuin-A to form colloidal calciprotein particles, which in turn can activate NLR protein 3 (NLRP3) via calcium-sensing receptors. This process facilitates the release of IL1β, thereby underscoring an alternative mechanism by which calcium is critically involved in the activation of NLRP3 [41]. In the initial phase of the immune response, the host detects pathogen-associated molecular patterns through pattern recognition receptors (PRRs). NLRs represent the largest class of PRRs, which can oligomerize and assemble into large signaling complexes, such as the inflammasomes. It is a cytosolic multiprotein complex that can activate pathways such as nuclear factor κB (NF-κB), mitogen-activated protein kinase (MAPK), and pyroptosis, leading to the release of pro-inflammatory cytokines, including tumor necrosis factor-α (TNF-α), IL1β, and IL18, thereby mediating a cascade of downstream immune-inflammatory responses [42–44].

**Fig. 6.**
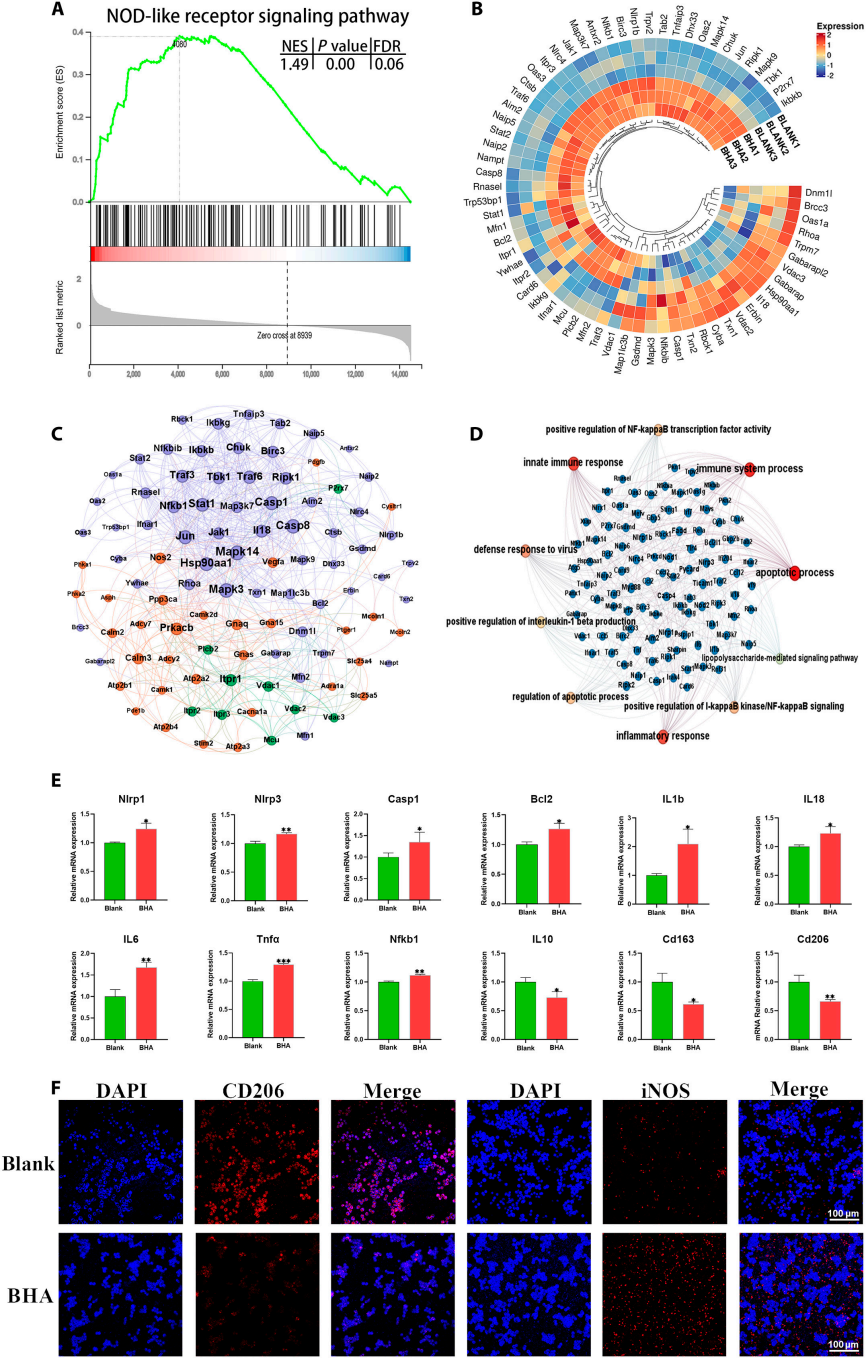
The macrophage toward to pro-inflammatory status in the BHA group. (A) GSEA result of the KEGG term NLR signaling pathway. |NES| > 1, *P* < 0.05, and FDR *q* <0.25 are considered significant. (B) Heatmap of differentially expressed genes in the NLR signaling pathway. (C) Interaction analysis of the calcium signaling pathway and NLR signaling pathway. Orange dots, genes involved in the calcium signaling pathway; purple dots, genes involved in the NLR signaling pathway; green dots, shared genes. (D) Interaction network showed close interaction between the NLR signaling pathway and immune response terms. (E) RT-qPCR results of NLR signaling pathway-related genes. (F) Representative immunofluorescence images showed up-regulated iNOS (M1 macrophage marker) and down-regulated CD206 (M2 macrophage marker) of macrophages in the BHA group. *P < 0.05; **P < 0.01; ***P < 0.001.

GSEA results revealed up-regulation of the NLR signaling pathway in the BHA group (Fig. 6A). The heatmap of differentially expressed genes within the NLR signaling pathway suggested that most involved genes were up-regulated in the BHA group (Fig. 6B). Interaction analysis showed that the NLR signaling pathway was closely regulated by the calcium signaling pathway, with numerous connected and shared genes (Fig. 6C). GO Biological Functional analysis of the differentially expressed genes in the NLR signaling pathway further revealed activation of a pro-inflammatory immune response, including processes such as inflammatory response, innate immune response, positive regulation of IL1β production, and positive regulation of IκB kinase/NF-κB signaling (Fig. 6D). RT-qPCR results further confirmed the up-regulation of NLR signaling pathway-related genes in the BHA group, including *Nlrp1*, *Nlrp3*, *Casp1*, *Bcl2*, *Il1b*, *Il18*, *Il6*, and *Tnfa*. Conversely, anti-inflammatory factors and M2 macrophage markers including *Il10*, *Cd163*, and *Cd206* were down-regulated (Fig. 6E). Immunofluorescence staining showed an up-regulation of iNOS (an M1 macrophage marker) and a down-regulation of CD206 (an M2 macrophage marker), further confirming the presence of a macrophage-mediated pro-inflammatory immune microenvironment in the BHA group (Fig. 6F).

In summary, BHA could interfere with fibrin clot formation by absorbing coagulation factors and releasing Ca^2+^. The increased Ca^2+^ binding within the fibrin clot led to the activation of the calcium signaling pathway. The activation of the calcium signaling pathway could further induce the activation of the NLR signaling pathway, which was closely interacted with the immune response, thereby establishing a macrophage-mediated pro-inflammatory immune microenvironment. This activated pro-inflammatory immune microenvironment in the BHA group would further suppress the periodontal “developmental engineering” process.

### IL4-BHA rescued BHA-mediated periodontal redevelopment failure via immunosuppression

The efficacy of the immunosuppression strategy on the periodontal redevelopmental process was further evaluated. Our previous study has summarized the key stages of periodontal developmental processes as epithelial induction, fiber formation, and mineralization [5]. Meanwhile, the mechanisms underlying the failure of BHA-mediated periodontal redevelopment were analyzed using high-throughput chip technology. Consequently, an IL4-doped BHA (IL4-BHA) was designed as an immunomodulatory biomaterial. IL4-BHA was implanted into rat periodontal defect model to assess the degree of redevelopment state and the regeneration of periodontal tissue.

**Fig. 7.**
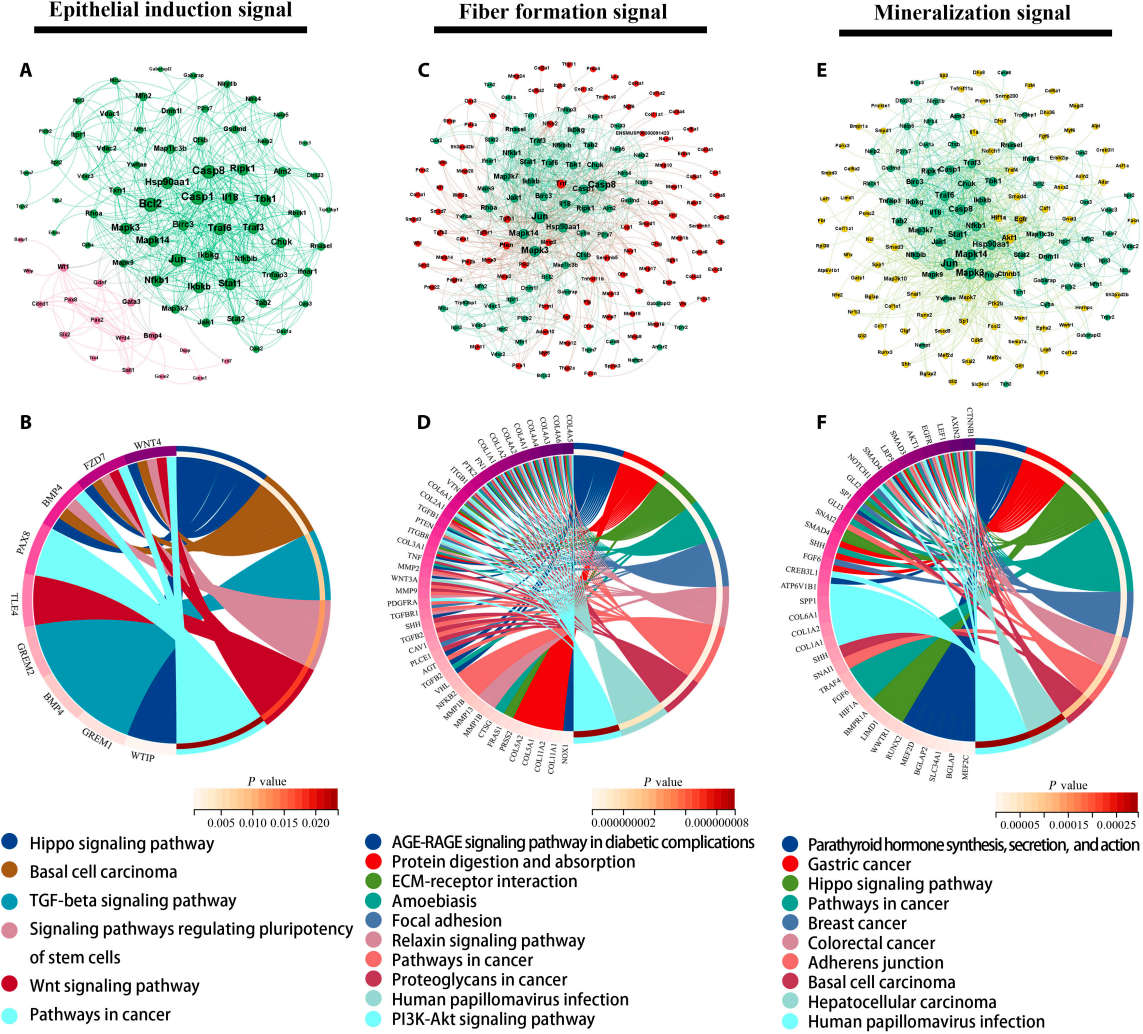
The development engineering potential regulated by macrophage-mediated immune microenvironment. (A) Interaction network showed weak interaction between the NLR signaling pathway and epithelial induction signal. (B) KEGG analysis of connected genes involved in epithelial induction signal. (C) Interaction network showed close interaction between the NLR signaling pathway and fiber formation signal. (D) KEGG analysis of connected genes involved in fiber formation signal. (E) Interaction network showed close interaction between the NLR signaling pathway and mineralization signal. (F) KEGG analysis of connected genes involved in mineralization. Green dots, genes involved in the NLR signaling pathway; pink dots, genes involved in epithelial induction signal; red dots, genes involved in fiber formation signal; yellow dots, genes involved in mineralization signal.

Regarding epithelial induction, only a small number of genes were found to connect the NLR signaling pathway with the epithelial-to-mesenchymal transition (EMT) process, indicating weak activation of epithelial induction in the BHA group (Fig. 7A). KEGG analysis of connected genes in downstream term was conducted (Fig. 7B). The transforming growth factor-β (TGF-β) signaling pathway was demonstrated to be involved in EMT during developmental morphogenetic events [45], but only a few genes were involved, including GREM1, BMP4, and GREM2. This suggested that epithelial induction was not robustly activated in the BHA group. ECM organization, encompassing fiber formation, degradation, and remodeling, was closely linked to the NLR signaling pathway, with numerous directly connected genes (Fig. 7C). Enriched KEGG terms included the phosphatidylinositol 3-kinase (PI3K)–Akt signaling pathway, ECM–receptor interaction, and focal adhesion (Fig. 7D). The PI3K–Akt signaling pathway is a critical signaling node during fibrosis [46], promoting the up-regulation of profibrogenic mediators and enhancing fibrotic activity. These results indicated that the macrophage-mediated immune microenvironment in the BHA group could induce collagen synthesis and degradation activity of fibroblasts, thus promoting tissue fibrosis activity. As for mineralization events, interaction analysis between the NLR signaling pathway and ossification term was conducted. As shown in Fig. 7E, genes involved in the 2 terms were closely related to each other, suggesting a close regulatory relationship. Enriched terms of connected genes included the Hippo signaling pathway (Fig. 7F), which was involved in osteoblast differentiation and played a pivotal role in maintaining the dynamic balance between bone regeneration and resorption. Thus, the macrophage-mediated immune microenvironment in the BHA group may promote osteoblast differentiation and maintain bone homeostasis.

In conclusion, the epithelial induction activity was not strongly activated in the BHA group. The ECM synthesis and degradation were notably activated, consistent with the fibrotic activity phenomenon in vivo, while the ossification could be enhanced in the BHA group by activating the Hippo signaling pathway.

**Fig. 8.**
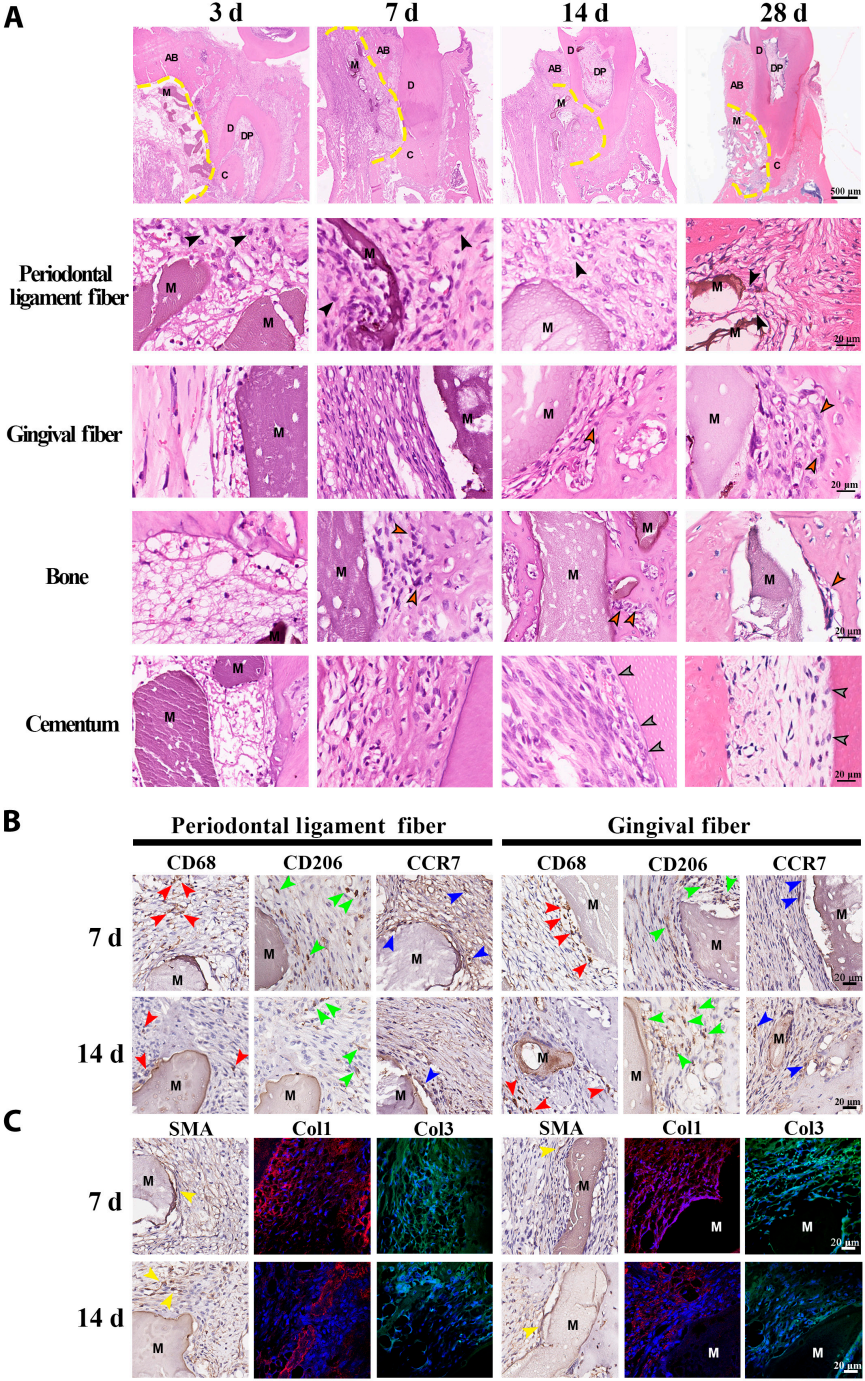
IL4-BHA-mediated periodontal redevelopment engineering. (A) Overview of redevelopment state and the detailed view of periodontal ligament fiber, gingival fiber, alveolar bone, and cementum in the IL4-BHA group. (B) The pro-inflammatory microenvironment was reversed in the IL4-BHA group. (C) ECM synthesis activity in periodontal ligament and gingival fiber. Black arrowhead, PDLCs; orange arrowhead, osteoblast; gray arrowhead, cementoblast-like cells; red arrowhead, CD68^+^ cells; green arrowhead, CD206^+^ cells; blue arrowhead, CCR7^+^ cells; yellow arrowhead, SMA^+^ cells; M, material; D, dentin; C, cementum; AB, alveolar bone; DP, dental pulp.

IL4 is a typical anti-inflammatory cytokine with the ability to induce the polarization of macrophages toward the M2 phenotype. The IL4-induced microenvironment demonstrated superior efficacy in promoting epithelial induction, fiber formation, and the mineralization of PDLCs [6]. Consequently, IL4 was selected for the modification of BHA. A PDA coating was applied to the BHA surface, as confirmed by SEM, FTIR, and XPS analyses. Immunofluorescence staining revealed the successful distribution and loading of IL4 on the BHA surface (Fig. S6). Following the implantation of IL4-BHA, the immune microenvironment was successfully shifted toward an M2-like profile, as indicated by an decreased ratio of CCR7-positive cells and CD206-positive cells at 7 and 14 d after surgery (Fig. 8B and Fig. S7). Within the periodontal ligament region, PDLCs migrated into the defect area and secreted collagen matrix, leading to the formation of a mature ECM structure (Fig. 8A). A distinct boundary between the blood fibrin clot and the outer gingival fibers was observed, effectively preventing early ingrowth of outer gingival fibroblasts (Fig. 8A). Additionally, the number of SMA-positive cells was low in the IL4-BHA group compared with the BHA group (Fig. S7), mitigating the risk of excessive collagen deposition, including COL1 and COL3 (Fig. 8C and Fig. S8). ALP-positive cells and OPN-positive cells were detected in the newly formed alveolar bone and adjacent collagen fibers at 28 d after surgery (Fig. S9B), indicating active osteogenesis. Cementoblast-like cells were also observed on the exposed dentin surface (Fig. 8A). Furthermore, picrosirius red staining (PR) staining revealed highly organized, parallel periodontal fibers extending from the newly formed alveolar bone to the root surface (Fig. S9C), suggesting superior cementum regeneration compared to the Blank and BHA groups.

**Fig. 9.**
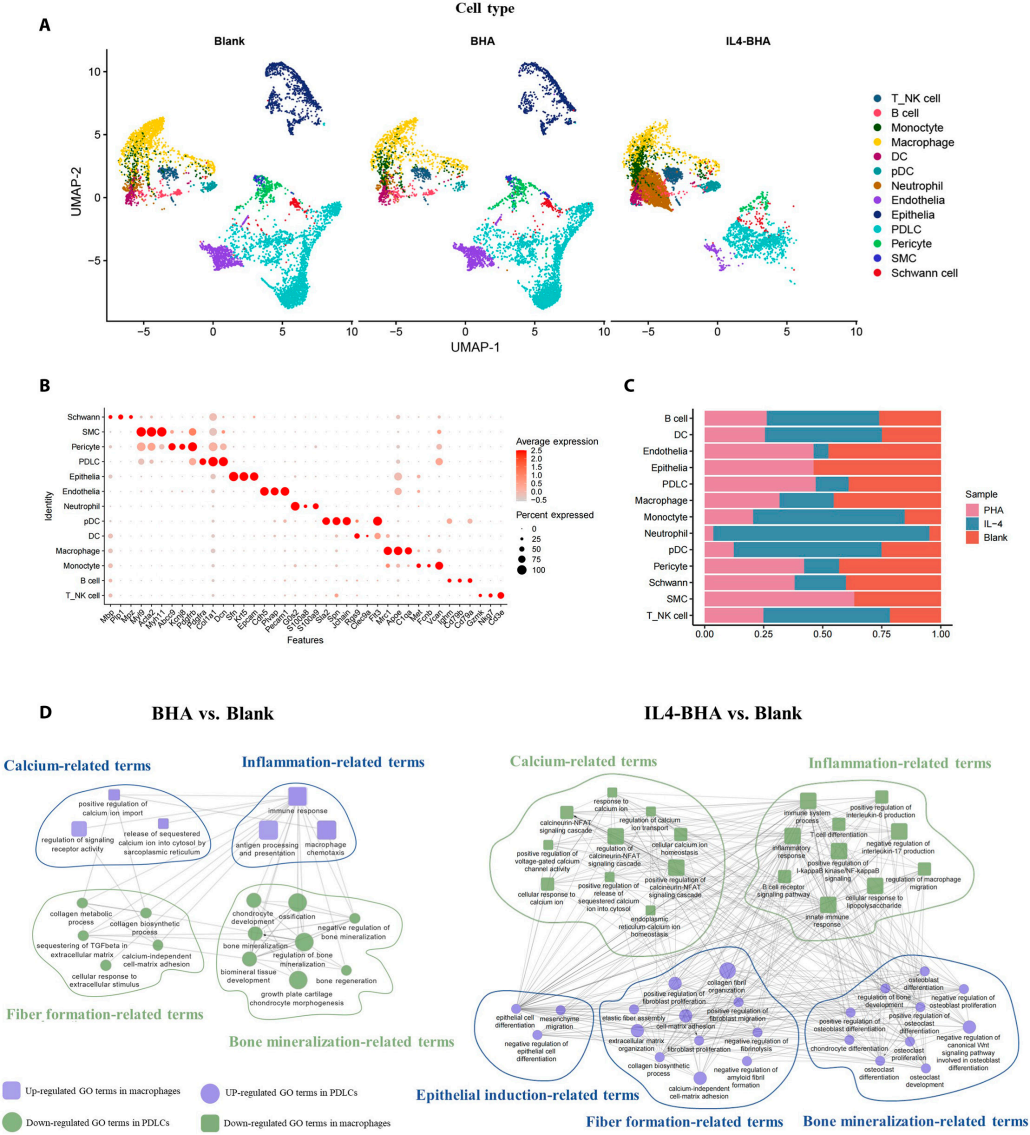
The assessment of periodontal redevelopmental capacity in vivo through scRNA-seq. (A) UMAP plot showed the clustering results. The colors represented the major cell types. (B) Dot plot showed the highly expressed marker genes in each major cell type. (C) Stacked histogram showed the percentages of the major cell types. (D) Relationship between the calcium–inflammation axis and the recurrence of periodontal key developmental events in BHA and IL4-BHA groups. Square dots, GO terms enriched by significantly different genes in macrophages; round dots, GO terms enriched by significantly different genes in PDLCs; purple dots, up-regulated GO terms; green dots, down-regulated GO terms.

To further investigate the effects of the calcium–NOD-like receptor–inflammation axis on recurring the periodontal developmental process in vivo, we implanted BHA and IL4-doped BHA into periodontal defects in rats. Seven days after implantation, we collected the newly formed tissue from the defects and performed scRNA-seq to assess the healing conditions in the BHA and IL4-BHA groups (Fig. 9A to C). The results indicated that, compared to the Blank group, macrophages in the BHA group exhibited an up-regulation of the calcium ions and inflammation-related pathways, while the activity related to key periodontal developmental events in PDLCs, such as fiber formation and mineralization, decreased (Fig. 9D). Conversely, compared to the Blank group, macrophages in the IL4-BHA group demonstrated a down-regulation of the calcium ions and inflammation-related pathways, while the key periodontal developmental events in PDLCs, including epithelial induction, fiber formation, and mineralization, were up-regulated (Fig. 9D).

In summary, the incorporation of IL4 effectively established an M2-like immune microenvironment by inhibiting the calcium–inflammation axis and stabilized the fibrin clot structure, thereby preventing the early migration of outer gingival fibroblasts. This allowed PDLCs to migrate and form an ECM, embedding Sharpey’s fibers into both the alveolar bone and cementoid. These processes recur critical events in periodontal development, thereby enabling IL4-BHA to rescue BHA-mediated periodontal redevelopment failure.

### Implications for the optimization of periodontal BHA based on developmental engineering strategies

The intricate functions of periodontal tissue in maintaining and supporting teeth rely on its complex structure, which poses important challenges for complete restoration. Historically, periodontal regeneration has focused on achieving multi-tissue regeneration, prompting extensive research into biocompatible materials capable of promoting the multidirectional differentiation of PDLCs and possessing multi-layered structures for diverse tissue repair. Despite the rigor of these studies, the clinical outcomes of these materials have not been entirely satisfactory. Recent advancements in developmental biology and regenerative medicine have opened new avenues for periodontal tissue regeneration. During organ development, tissues, cells, and signaling molecules engage in a highly ordered assembly process, resulting in organs with complex structures. However, the regenerative capabilities of cells during the healing processes are considerably more limited. Traditional approaches in regenerative medicine have predominantly concentrated on understanding and mimicking the process of tissue repair. In contrast, harnessing and reconstructing the developmental programs of organs represents a pivotal breakthrough and a promising future direction in regenerative medicine. The clinical application of amelogenin, a protein involved in periodontal formation during development, has demonstrated improved therapeutic outcomes in certain cases, suggesting potential clinical support for developmental engineering strategies. Accordingly, we propose that effective modification of BHA should be based on the concept of developmental engineering. Rather than assessing modification strategies solely by their ability to promote the multidirectional differentiation of PDLCs, as done in previous studies, we advocated for evaluating these strategies based on their capacity to induce a redevelopmental state. We further found that the key difference between the microenvironments of developmental and healing processes lay in the shift of the immune microenvironment, and the induction of redevelopment status may be regulated by immune microenvironment.

Our research demonstrated that BHA-mediated periodontal tissue healing was often accompanied by an excessive inflammatory response, primarily regulated by the calcium–NOD-like receptor–inflammation axis. Application of IL4 to modulate this inflammation markedly improved periodontal tissue repair. However, while our study preliminarily established the feasibility of immunosuppression in periodontal redevelopment using IL4-coated BHA in vivo, further research is necessary to elucidate the dose-dependent effects of such immunomodulatory agents and the underlying mechanisms. Previous studies have confirmed that immune microenvironment regulation is beneficial for periodontal tissue healing [6]. Our study provides mechanistic insights into the regulation of tissue regeneration by the immune microenvironment from a developmental perspective. In this research, we mainly focused on macrophages given their crucial role in immune response activation and periodontal regeneration. However, this does not imply that other immune cells, such as lymphocytes, neutrophils, and mast cells, are not important. The underlying relationship of immune response and redevelopment requires further investigation.

Although modifying the immune environment can partially revert defective periodontal tissue to a developmental state, achieving complete periodontal regeneration remains a distant goal. This suggests that, beyond microenvironmental differences, the disparities in cell populations between developmental and healing phases warrant further attention. Periodontal development is predominantly driven by dental follicle cells, whereas periodontal repair is mainly mediated by PDLCs, which reside in the adult periodontal ligament. Our prior research revealed that the macrophage-mediated immune microenvironment failed to fully induce the redevelopmental state of PDLCs [5]. Moreover, clinical trials have further corroborated that PDLCs are inadequate for developmental engineering. Thus, it is evident that successful application of developmental engineering strategies cannot rely solely on PDLCs. Future efforts in periodontal developmental engineering should focus on how to effectively leverage cells involved in the developmental phase.

The results of this study demonstrated, through in vitro RNA-seq of macrophages and in vivo single-cell transcriptomic sequencing of periodontal defect, that the BHA group caused the failure of periodontal redevelopment via the calcium–NOD-like receptor–inflammation axis, while IL4-BHA could partially reverse this process. Future investigations may use gene conditional knockout models and spatial transcriptomics to further clarify the crucial target cell subpopulations, essential genes, and regulatory molecules within this regulatory axis.

## Conclusion

Developmental engineering may represent the most promising strategy for achieving periodontal tissue regeneration. Our results indicated that neither the Blank group nor the BHA group were able to fully recur periodontal developmental events. The pro-inflammatory microenvironment observed in the BHA group was the primary factor contributing to the suboptimal outcomes of “developmental engineering”. This microenvironment was activated via the calcium–NOD-like receptor–inflammation axis, which played a critical role in the observed redevelopment failure. The addition of IL4 was able to partially rescue this failure through its immunosuppressive effects. This study has pioneered a strategy for the creation of advanced periodontal regenerative materials, offering a potential pathway for the further application of development-inspired periodontal tissue engineering strategies.

## Data Availability

The data are available from the corresponding author on reasonable request.
