# Supplementary material for: Evolution of Biological Hydroxyapatite Modification Strategy: Anti-Inflammation Approach Rescues the Calcium–NOD-Like Receptor–Inflammation Axis-Mediated Periodontal Redevelopment Failure
Source: Biomater Res. 2025 Feb 26;29:0131. doi: 10.34133/bmr.0131 (PMC11862812; doi:10.34133/bmr.0131)
Supplement: Supplementary 1 — Figs. S1 to S9 Table S1 [file bmr.0131.f1.docx]

**
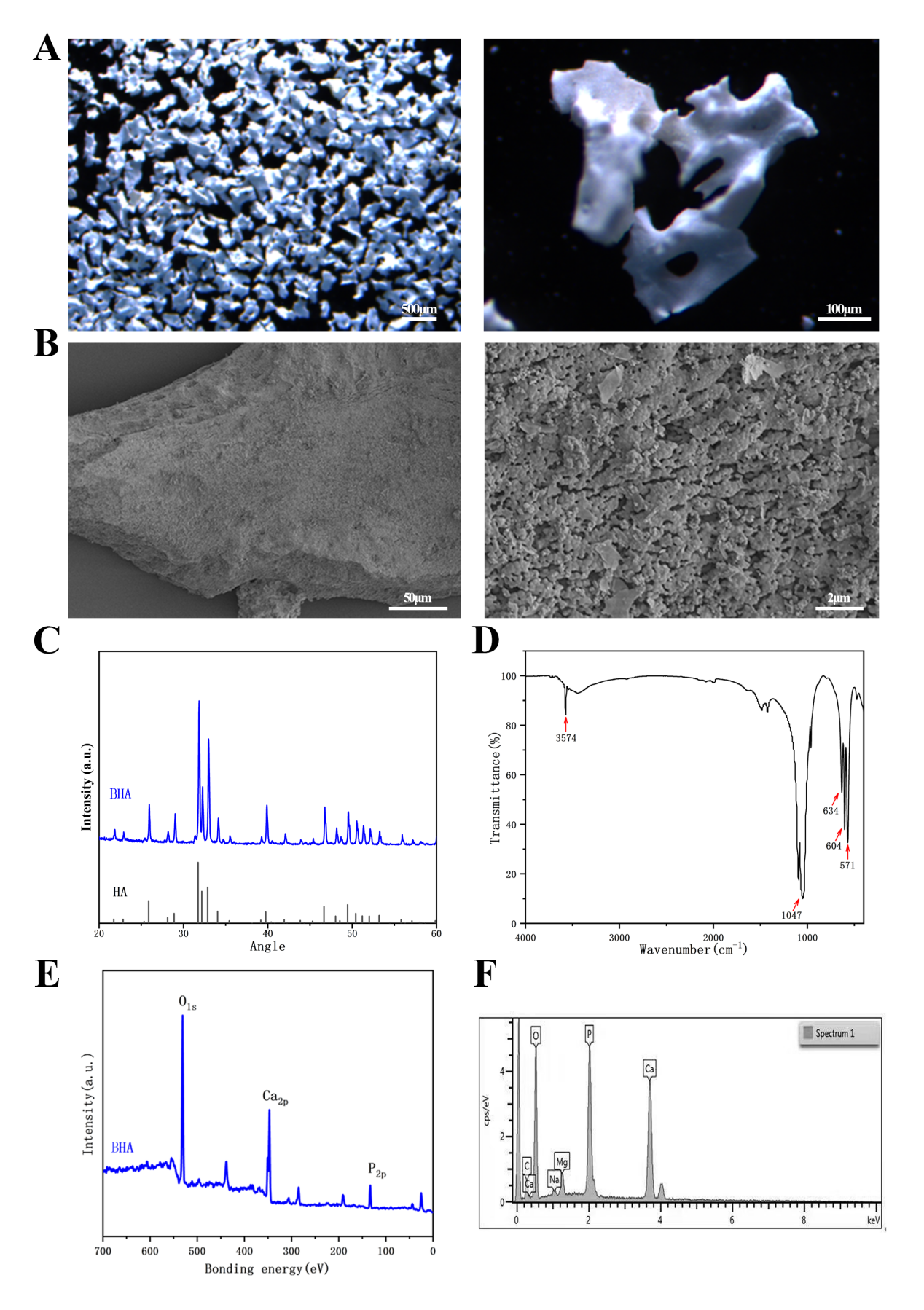
**

**Supplementary Figure1. Characterization of BHA.** A) Macroscope images of BHA particles. B) The SEM image showed biomimetic microporous structure of BHA particle. C) The XRD result showed the crystal structure of BHA was consistent with the hydroxyapatite. D-F) The FTIR, XPS and EDS results confirmed the element component of BHA.

**
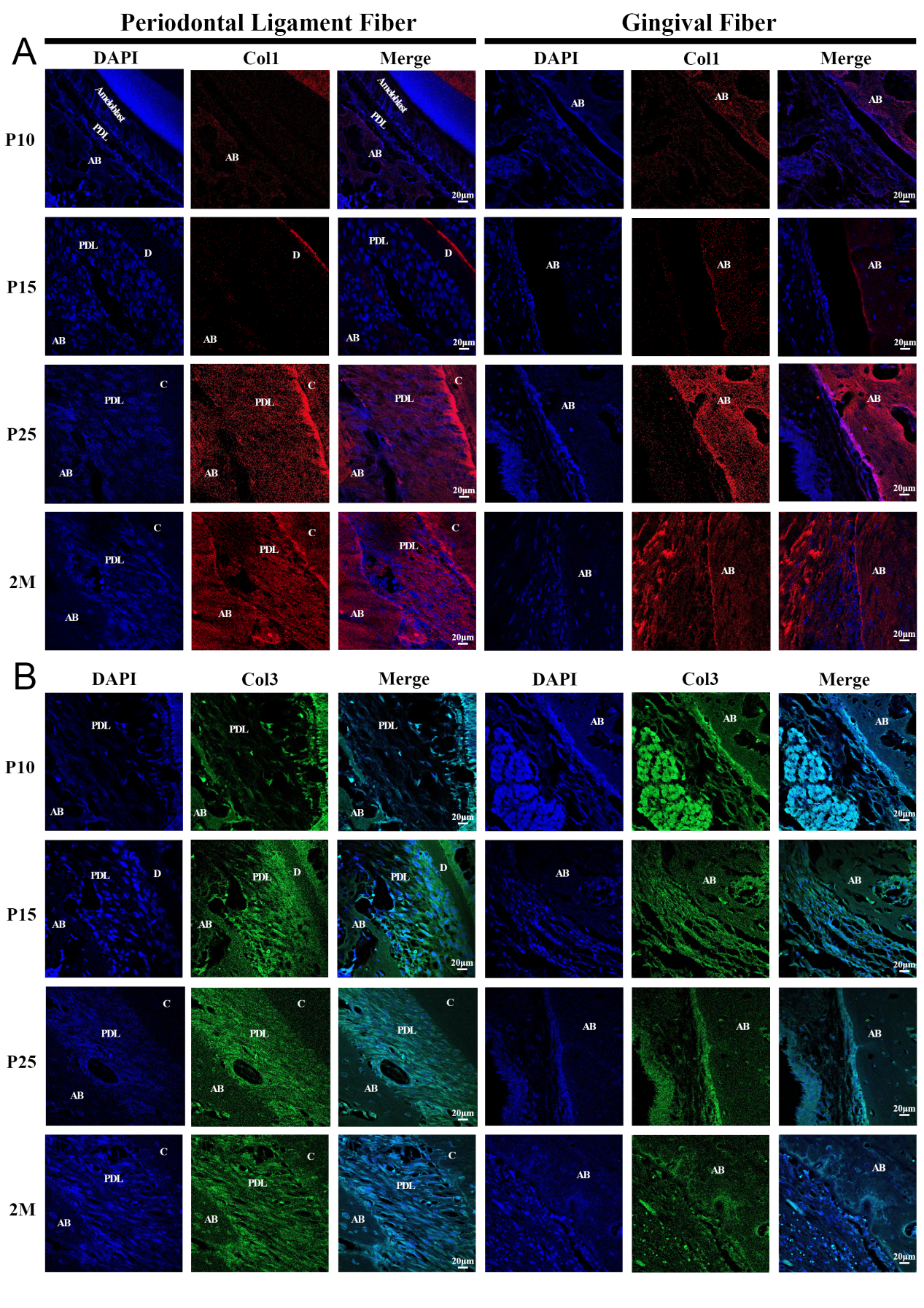
**

**Supplementary Figure2. Immunofluorescent staining of Col1 and Col3 during developmental process.** A) Immunofluorescent staining of Col1 in developmental process. B) Immunofluorescent staining of Col3 in developmental process. AB, alveolar bone; D, dentin; PDL, periodontal ligament; C, cementum.
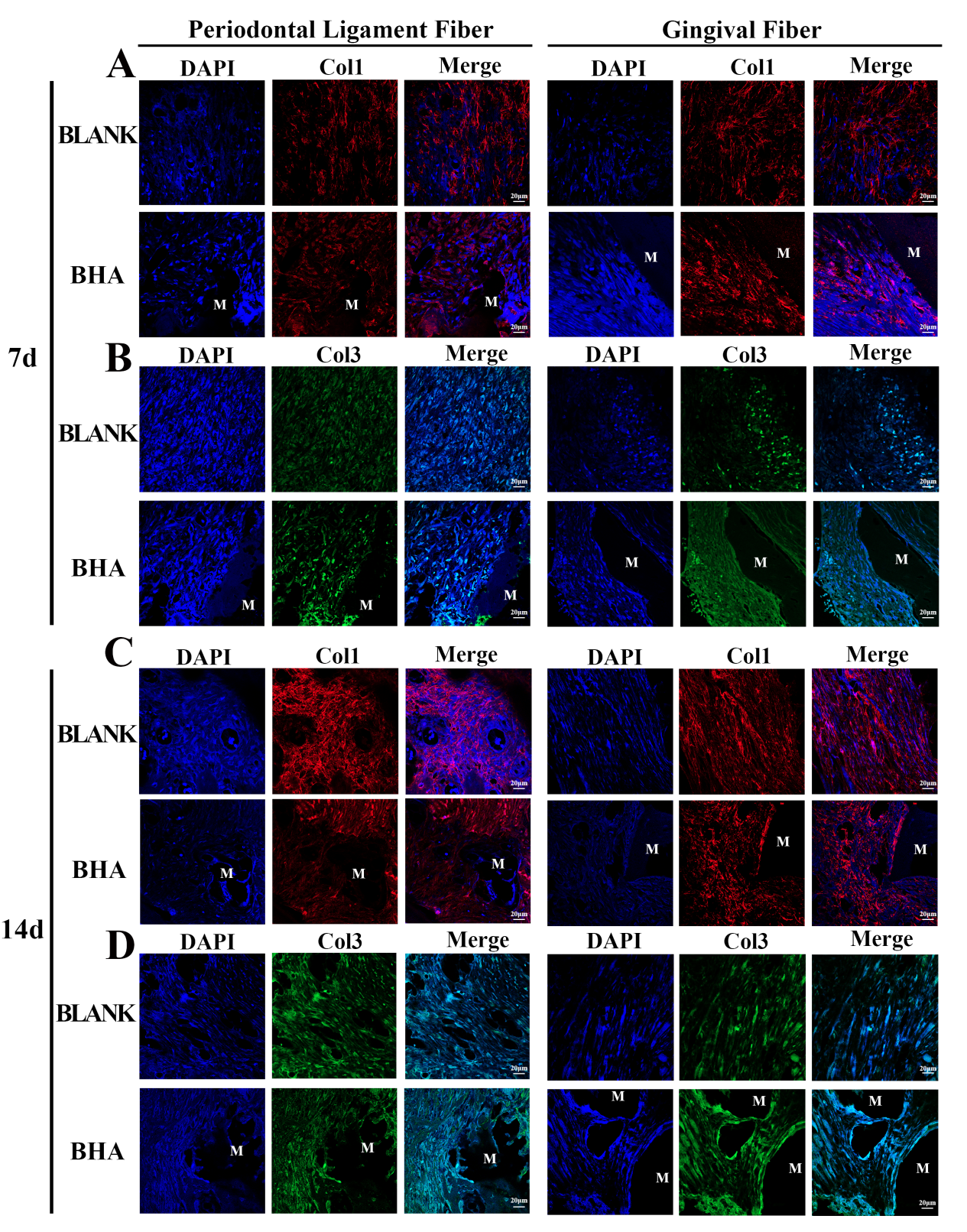


**Supplementary Figure3. Immunofluorescent staining of Col1 and Col3 at 7 and 14 days post-surgery in Blank and BHA group.** A) Immunofluorescent staining of Col1 at 7 days post-surgery. B) Immunofluorescent staining of Col3 at 7 days post-surgery. C) Immunofluorescent staining of Col1 at 14 days post-surgery. D) Immunofluorescent staining of Col3 at 14 days post-surgery. M, material.

**
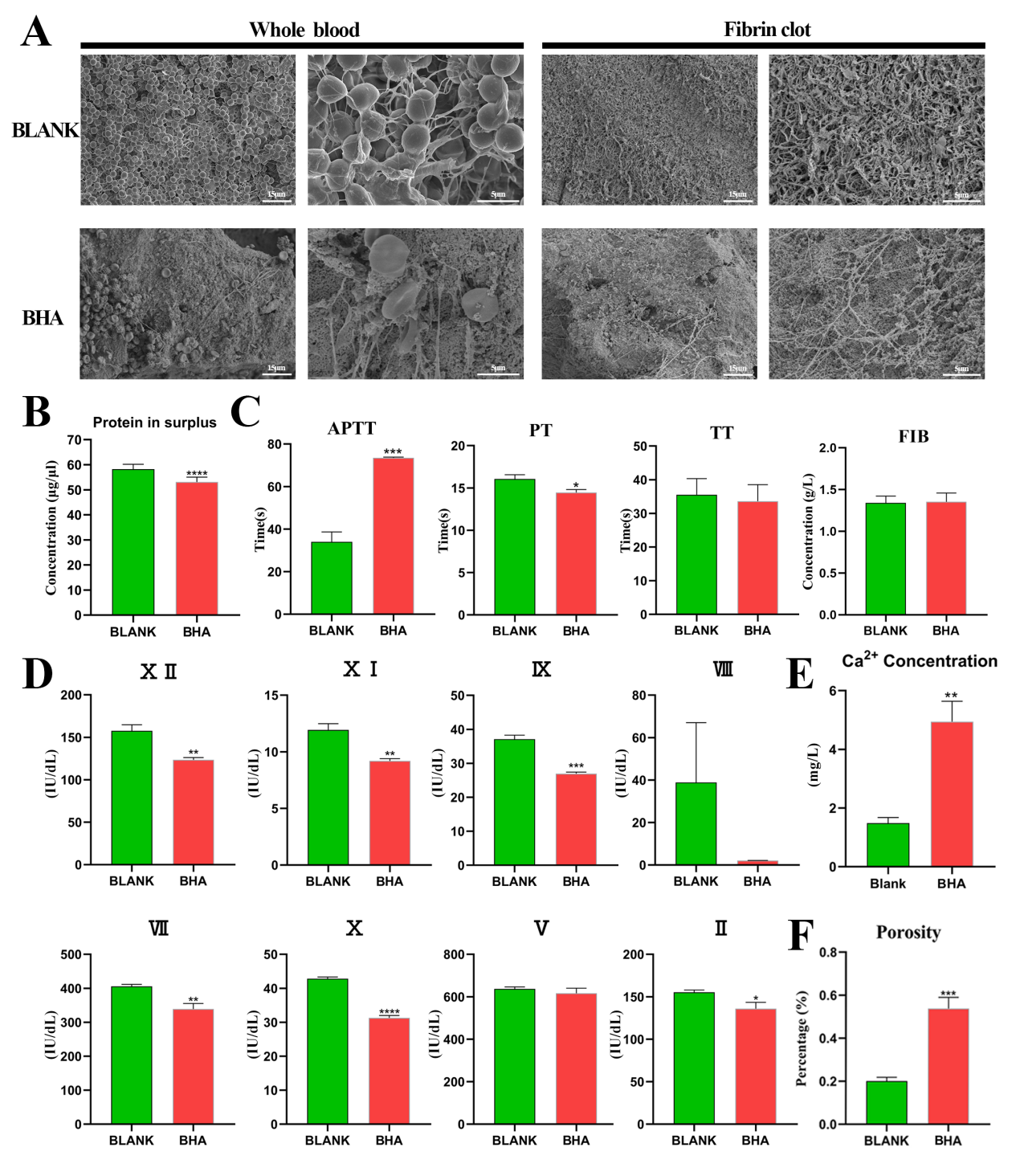
**

**Supplementary Figure 4. The effect of BHA on fibrin clot formation.** A) The SEM result of blood clot and fibrin clot. B) Residual protein concentration in plasma after BHA adsorption. C) The APTT, PT, TT and FIB results of normal plasma and BHA-adsorbed plasma. D) The concentration of clotting factors in normal plasma and BHA-adsorbed plasma. E) Residual calcium concentration in BHA co-worked plasma was increased than normal plasma. F) Semi-quantification of fibrin networks’ porosity. **p* < 0.05; ***p* < 0.01; ****p* < 0.001; *****p* < 0.0001.

**
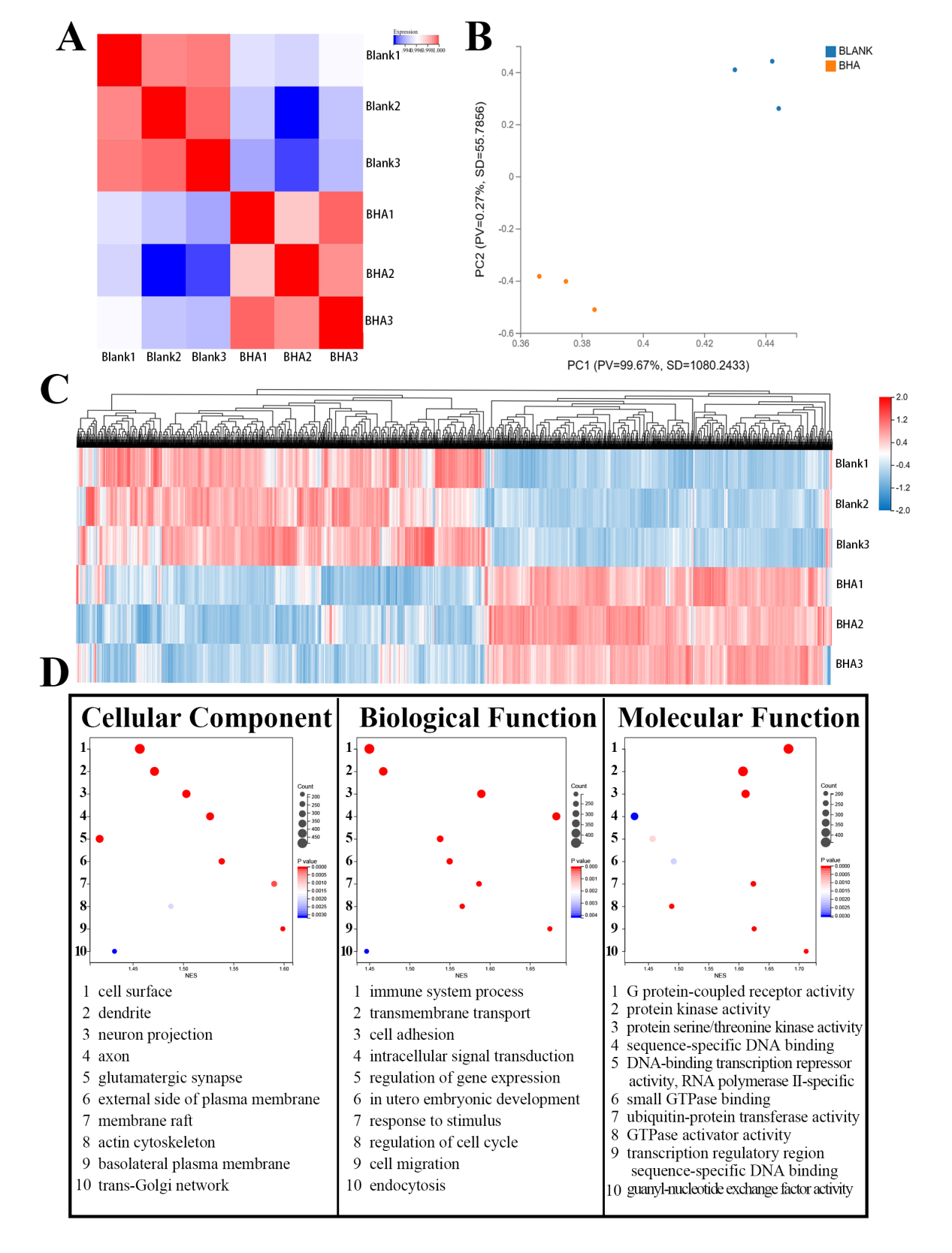
**

**Supplementary Figure 5. Overview of RNA-seq result of macrophages cultured by fibrin clot.** A) Correlation analysis of Blank and BHA group. B) Principal Component Analysis (PCA) result of Blank and BHA group. C) Heatmap of differentially expressed genes in Blank and BHA group. D) GSEA revealed top 10 up-regulated GO terms in BHA group, |NES|>1, p-val <0.05 and FDR q-val <0.25 are considered significant.

**
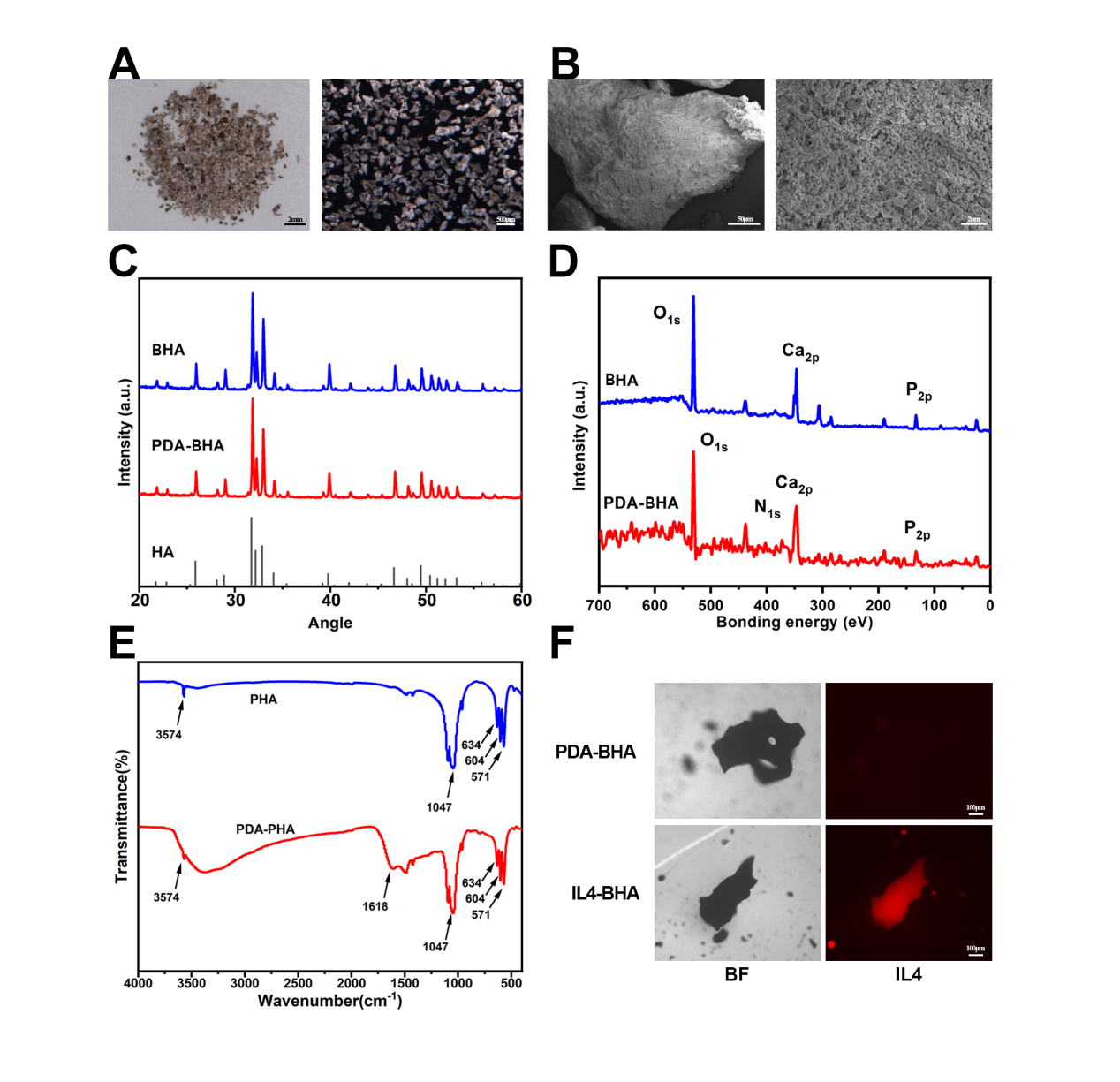
**

**Supplementary Figure 6. Preparation and characterization of IL4-BHA.** A-B) Macroscope and SEM image of PDA coated BHA. C) The XRD result showed no crystal structure changes in PDA coated BHA. D) The XPS result confirmed the N_1s_ peak in PDA coated BHA. E) The FTIR result showed the benzene ring in PDA coated BHA. F) The immunofluorescence staining showed the loading of IL4 on the surface of BHA particles.

**
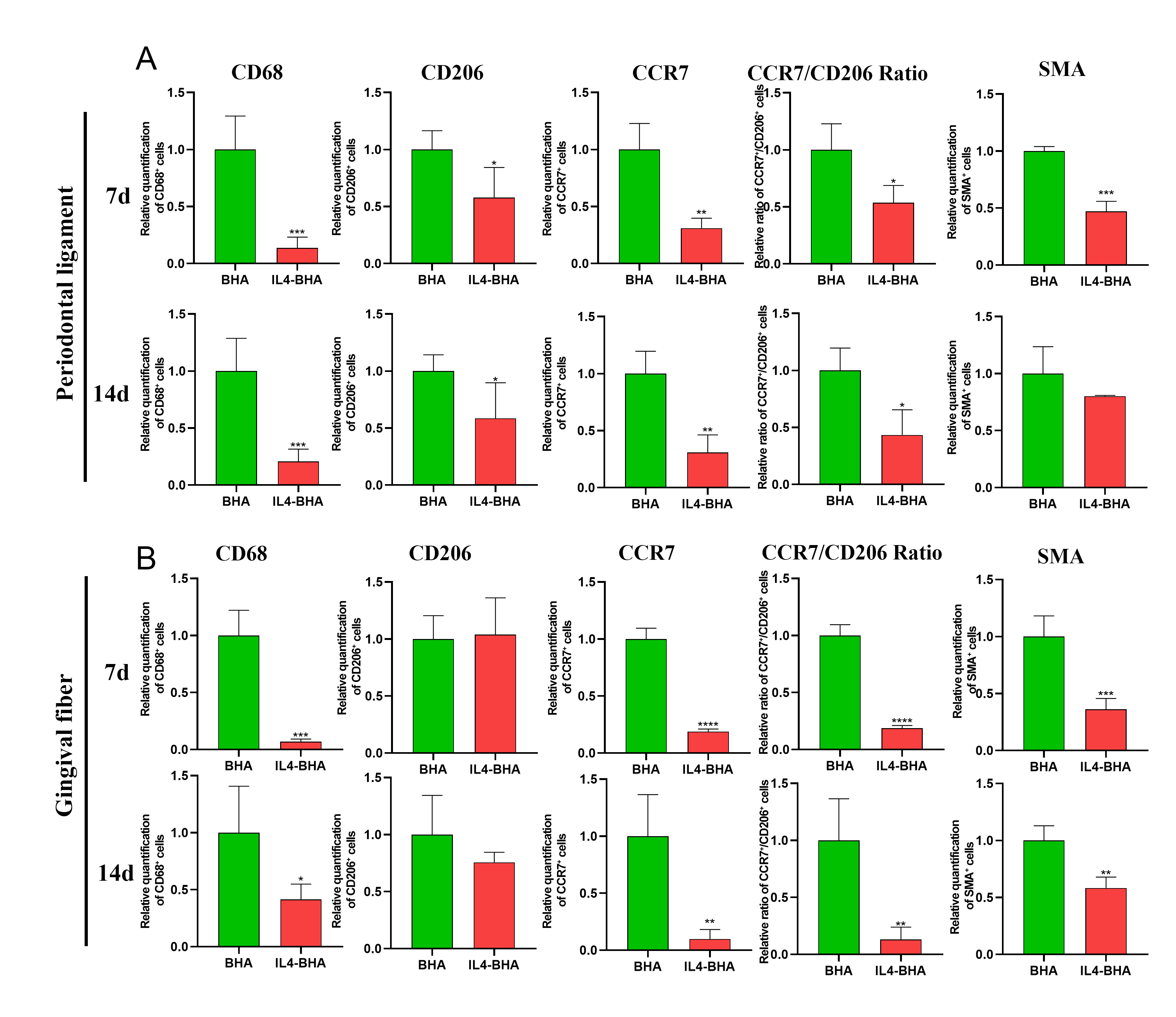
Supplementary Figure 7. Semi-quantification of macrophage markers and SMA at 7 and 14 days post-surgery.** A）Semi-quantification of macrophage markers (CD68^+^, CD206^+^, CCR7^+^ ) CCR7^+^/CD206^+^ ratio and SMA in BHA and IL4-BHA group in the periodontal ligament area. B）Semi-quantification of macrophage markers (CD68^+^, CD206^+^, CCR7^+^) CCR7^+^/CD206^+^ ratio and SMA in BHA and IL4-BHA group in outer gingival fiber area.

**
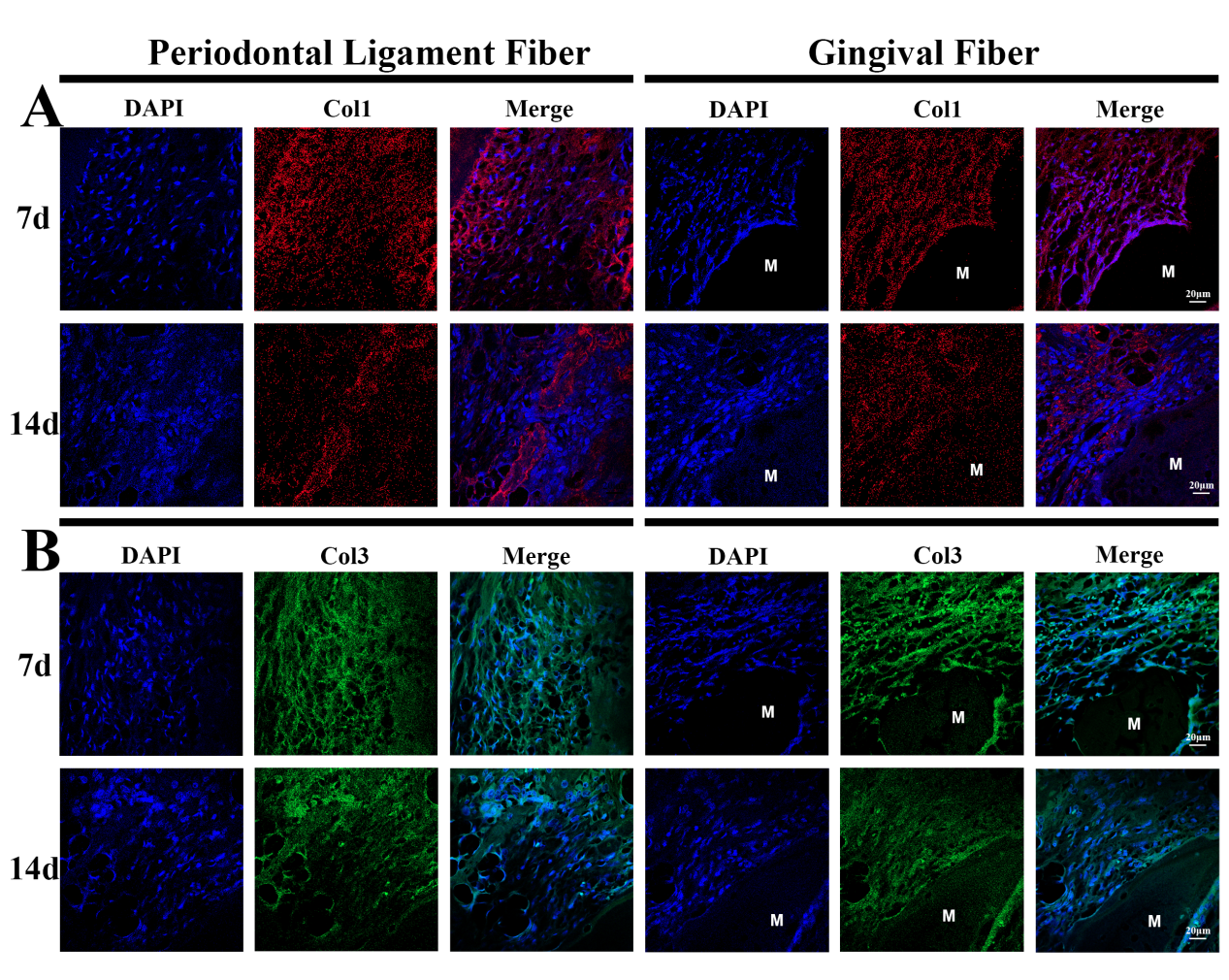
**

**Supplementary Figure 8. Immunofluorescent staining of Col1 and Col3 at 7 and 14 days after surgery in IL4-BHA group.** A) Immunofluorescent staining of Col1 at 7 and 14 days post-surgery. B) Immunofluorescent staining of Col3 at 7 and 14 days post-surgery. M, material.


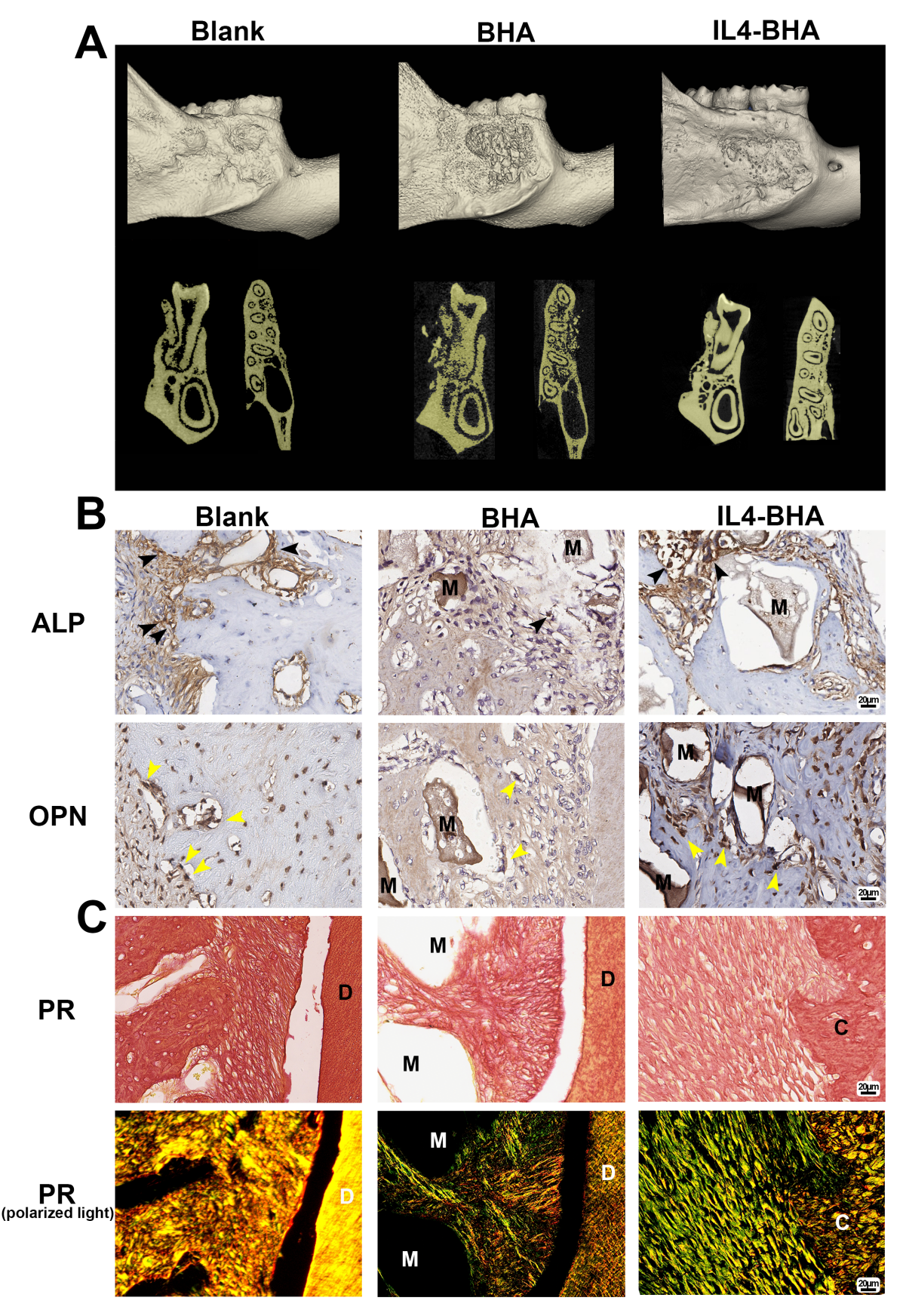


**Supplementary Figure 9. Multi-tissue regenerative effect at 28 days post-surgery.** A) Three-dimensional reconstruction of the micro-CT showed the overview of the periodontal regeneration outcome. B) The bone formation effect was evaluated using ALP and OPN IHC staining. C) The fiber regeneration was evaluated using PR staining under non-polarized/polarized light microscopy. Black arrow head, ALP^+^ cells; Yellow arrow head, OPN^+^ cells; M, material; D, dentin; C, cementum.

Supplementary Table1.RT-qPCR primers applied in this study

| Genes | Primer sequences (5’-3’) | |
| --- | --- | --- |
| g-Gapdh | Forward:  Reverse: | TCAGCAATGCCTCCTGCAC  TCTGGGTGGCAGTGATGGC |
| m-Cacna1a | Forward: | AGGCACCCTTTTGATGGAG |
|  | Reverse: | GCGGATGTAGAAACGCATTC |
| m-Atp2b1 | Forward: | CCATGCCGAAAGGGAGTTGC |
|  | Reverse: | CGGAGGGCTGGAGTTGCGTT |
| m-Atp2b4 | Forward: | AAGAAAATGATGAAGGACAACAAC |
|  | Reverse: | ATTGCGTACCATATTATCTCGGTC |
| m-TRPV2 | Forward: | GGCATACACAGAAGGCTCCA |
|  | Reverse: | CCGGAATCCCTGTCAATCTG |
| m-Trpm7 | Forward: | ACCCTCACAGATGTCTTCCAG |
|  | Reverse: | CATCTGAGTATTTTGTGGCAAG |
| m-Calm2 | Forward: | TTTATTTGCCTTTTCTTTGTTTG |
|  | Reverse: | TGGATTTGAGGCAAGTTGTG |
| m-Calm3 | Forward: | GATTAATGAGGTGGATGCTG |
|  | Reverse: | CATCTCCATCAATGTCGGCC |
| m-NOS2 | Forward: | TGCTGTTCTCAGCCCAACAA |
|  | Reverse: | GAACTCAATTGGCATGAGGCA |
| m-NLRP1 | Forward: | TGGCACATCCTAGGGAAATC |
|  | Reverse: | TCCTCACGTGACAGCAGAAC |
| m-NLRP3 | Forward: | GAGCTGGACCTCAGTGACAATGC |
|  | Reverse: | ACCAATGCGAGATCCTGACAACAC |
| m-casp1 | Forward: | TTACTGCTATGGACAAGGCACGGG |
|  | Reverse: | ATTGAGGGCAAGACGTGTACGAGTG |
| m-bcl2 | Forward: | AGAGCGTCAACAGGGAGATG |
|  | Reverse: | GATGCCGGTTCAGGTACTCAG |
| m-Nfkb1 | Forward: | TCCACTGTCTGCCTCTCTCGTC |
|  | Reverse: | GCCTTCAATAGGTCCTTCCTGC |
| m-IL10 | Forward: | GAGAAGCATGGCCCAGAAATC |
|  | Reverse: | GAGAAATCGATGACAGCGCC |
| m-CD163 | Forward: | ACATCATGGCACAGGTCAC |
|  | Reverse: | TGAGGAAACTGTAAGTCGCTG |
| m-CD206 | Forward: | AGACGAAATCCCTGCTACTG |
|  | Reverse: | CACCCATTCGAAGGCATTC |
| m-IL1B | Forward: | TGGAGAGTGTGGATCCCAAG |
|  | Reverse: | GGTGCTGATGTACCAGTTGG |
| IL18 | Forward: | GATGGTCTTGGTCCC |
|  | Reverse: | GATGGTCTTGGTCCC |
| m-IL6 | Forward: | ATAGTCCTTCCTACCCCAATTTCC |
|  | Reverse: | GATGAATTGGATGGTCTTGGTCC |
| m-TNFα | Forward: | GATCGGTCCCCAAAGGGATG |
|  | Reverse: | GGTTTGCTACGACGTGGGC |
